# *Helicobacter pylori* Inhibition, Gastritis Attenuation, and Gut Microbiota Protection in C57BL/6 Mice by *Ligilactobacillus salivarius* NCUH062003

**DOI:** 10.3390/microorganisms12122521

**Published:** 2024-12-07

**Authors:** Junyi Li, Xiaoyan Xu, Shiyu Yang, Kui Liu, Min Wu, Mingyong Xie, Tao Xiong

**Affiliations:** 1 State Key Laboratory of Food Science and Resources, Nanchang University, No. 235 Nanjing East Road, Nanchang 330047, China; 352313318007@email.ncu.edu.cn (J.L.);; 2 School of Food Science & Technology, Nanchang University, No. 235 Nanjing East Road, Nanchang 330047, China

**Keywords:** *Helicobacter pylori*, antibacterial activity, inflammatory effect, gut microbiota, *Ligilactobacillus salivarius* NCUH062003

## Abstract

*Helicobacter pylori* (*H. pylori*), one of the most prevalent pathogenic bacteria worldwide, is the leading cause of gastritis, gastric intestinal metaplasia, and gastric cancer. Antibiotics, the conventional treatment for eliminating *H. pylori*, often lead to severe bacterial resistance, gut dysbiosis, and hepatic insufficiency and fail to address the inflammatory response or gastric mucosal damage caused by *H. pylori* infection. In this study, based on 10-week animal experiments, two models of *L. salivarius* NCUH062003 for the prophylaxis and therapy of *H. pylori* infection in C57BL/6 mice were established; a comprehensive comparative analysis was performed to investigate the anti-*H. pylori* effect of probiotics, the reduction in inflammation, and repair of gastric mucosal damage. ELISA, immunohistochemistry, and pathology analyses showed that NCUH062003 decreased the expression of pro-inflammatory cytokine interleukins (IL-1β, IL-6) and myeloperoxidase (MPO) and reduced neutrophil infiltration in the gastric mucosa lamina propria. Immunofluorescence and biochemical analysis showed that NCUH062003 resisted gastric epithelial cell apoptosis, increased the level of superoxide dismutase (SOD) in gastric mucosa, and promoted the expression of tight junction protein ZO1 and Occludin. In addition, through high-throughput sequencing, in the probiotic therapy and prophylactic mode, the diversity and composition of the gut microbiota of HP-infected mice were clarified, the potential functions of the gut microbiota were analyzed, the levels of short-chain fatty acids (SCFAs) were measured, and the effects of *L. salivarius* NCUH062003 on the gut microbiota and its metabolites in HP-infected mice treated with amoxicillin/metronidazole were revealed. This study provides functional strain resources for the development and application of microbial agents seeking to antagonize *H. pylori* beyond antibiotics.

## 1. Introduction

*Helicobacter pylori* (*H. pylori*), identified in 1982 by Robin Warren and Barry J. Marshall [1], has been listed as a Class I carcinogen both by the World Health Organization and the International Agency for Research on Cancer since 1994, and was further classified a definitive carcinogen in 2021 in the 15th Report on Carcinogens published by the U.S. [2]. Currently, the prevalence of *H. pylori* infections varies across continents, with a global average infection rate of 44.5% [3]. The virulence factors secreted by *H. pylori*, such as lipopolysaccharide, cytotoxin-associated proteins, and vacuolar toxins, act directly on the gastric mucosa and stimulate the secretion of high amounts of inflammatory chemokines by mucosal cells, neutrophils, and macrophages, thus inducing a severe mucosal inflammatory response [4]. In addition, excessive production of oxygen free radicals by activated inflammatory cells through respiration leads to mitochondrial damage and gastric mucosal epithelial cell protein denaturation, resulting in gastric mucosal destruction mediated by oxidative stress, further leading to gastrointestinal disorders [5]. Therefore, an important consensus published in Kyoto, Toronto, China, and Maastricht on the therapy of *H. pylori* infection unanimously agreed that *H. pylori* eradication is necessary [6,7,8,9,10].

The gut microbiome resembles a vital organ of the human body and plays a key role in host health, influencing a range of processes from nutrient metabolism to immune function and behavior [11]. The traditional approach to eradicating *H. pylori* involves antibiotic therapies such as dual therapy (proton pump inhibitor (PPI) and amoxicillin), triple therapy (PPI, amoxicillin, and metronidazole), and bismuth quadruple therapy [12,13]. The side effects of antibiotic therapy are pronounced, including decreased eradication rates, increased therapy-emergent adverse events, and gastrointestinal microbiota dysbiosis [14], and repeated eradication therapy also tends to cause antibiotic resistance problems [15]. The WHO report showed that the resistance rates for clarithromycin, metronidazole, and levofloxacin are 14–34%, 20–38%, and 30–38%, respectively [16,17]. Moreover, an experimental animal study confirmed that *H. pylori* infection can cause significant changes in the gut microbiota of the uninflamed distal gastrointestinal tract [18]. According to Holistic Integrative Medicine (HIM), therapy for *H. pylori* should consider the physiology and pathology of the gastrointestinal tract along with the microecological environment [19]. Therefore, new alternatives are needed to combat *H. pylori*, addressing antibiotic resistance and gut microbiota damage.

In recent years, the emergence of microecology and related research has provided new possibilities for this purpose. Probiotics are a safe and simple alternative to antibiotic therapy for treating *H. pylori* infection [20]. And probiotic supplementation is considered one of the most promising ways to treat asymptomatic patients with *H. pylori* infection [21]. Probiotics refer to live microorganisms, the intake of which in sufficient quantities is beneficial to the health of the host [22,23]. Numerous in vitro and in vivo studies have demonstrated that probiotic strains, e.g., *B. lactis* BB12, *E. faecium* TM39, *L. casei* L26, *L. fermentum* MN-LF23, *L. gasseri* SBT2055, *L. gasseri* LG21, *L. johnsonii* No. 1088, and *L. rhamnosus* JS-SZ-2-1, have an excellent antagonistic ability against *H. pylori* [24,25]. Among these, *L. gasseri* LG21, screened by Meiji Dairy in Japan, is one of the most widely used probiotic strains. It also has a certain inhibitory effect on *H. pylori* strains tolerant to clarithromycin and has been successfully added to the LG21 series of yogurts [24]. Meanwhile, as stated in the 5th European Maastricht V Consensus and the 6th Chinese Consensus for *H. pylori* infection, probiotic preparations could serve as an adjunctive therapy for routine *H. pylori* eradication therapies [8,10].

**Figure 1:**
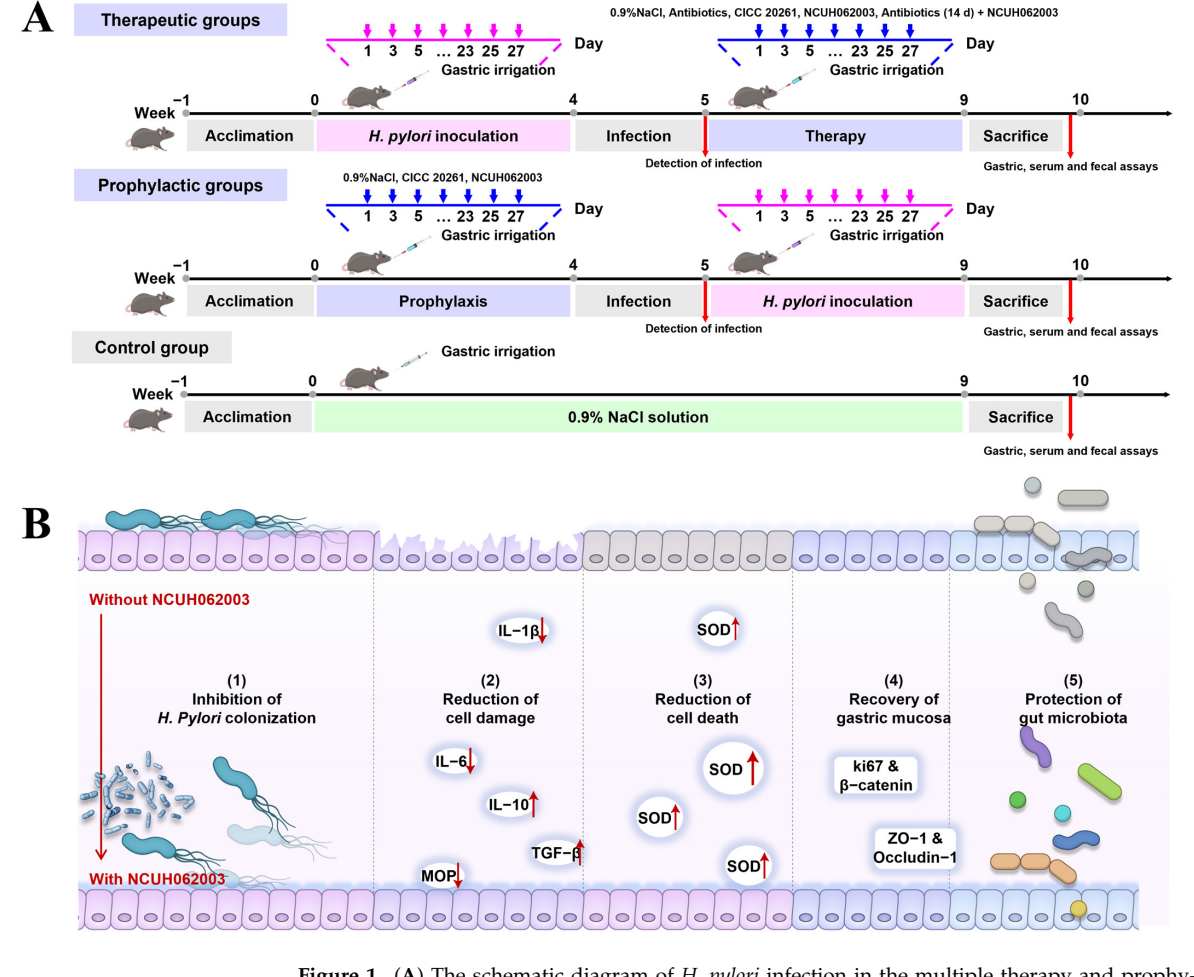
(**A**) The schematic diagram of *H. pylori* infection in the multiple therapy and prophylaxis processes conducted on C57BL/6 mice. (**B**) Schematic diagram showing the mechanisms of *L. salivarius* NCUH062003. A total of 9 groups were formed, with 8 mice in each group.

In the pre-experimental phase of our study, *Ligilactobacillus salivarius* NCUH062003, a probiotic strain screened from 50 lactic acid bacteria strains isolated from the oral cavity and gut of infants, stood out in a comprehensive in vitro evaluation of eight assays related to antagonism against *H. pylori* [26]. *L. salivarius* NCUH062003 demonstrated high gastric acid tolerance, rapid proliferation, superior anti-*H. pylori* activity, and high adhesion to gastric adenocarcinoma cells [26]. In gastric cell experiments, the strain exhibited a dual role in the exclusion and displacement of *H. pylori* colonization [26], which is rare among probiotics. Therefore, in this study, we performed mice experiments to verify the hypothesis in vivo that positions *L. salivarius* NCUH062003 as a potentially superior alternative to existing treatments. Prophylaxis and therapy for *H. pylori* infections in C57BL/6 mice (Figure 1A) were studied. *H. pylori* clearance, inflammation reduction, and gastric damage repair were analyzed. In addition, we analyzed the diversity and composition of the gut microbiota, predicted the potential functions of the gut microbiota, and determined the levels of short-chain fatty acids (SCFAs) to investigate the effects of *L. salivarius* NCUH062003 and its metabolites on the gut microbiota in HP-infected mice in therapy and prophylactic modes (Figure 1B).

## 2. Materials and Methods

### 2.1. Experimental Strains and Culture Conditions

*L. salivarius* NCUH062003 (abbreviation: LS03) is an antagonistic *H. pylori* strain pre-screened from the oral cavity of healthy newborns [26]. *L. plantarum* CICC 20261 (abbreviation: LP61) purchased from the China Center of Industrial Culture Collection (CICC) was used as the reference strain. The above-mentioned strains were stored in 25% (*v*/*v*) glycerol at −80 °C and incubated in MRS (Oxoid, Basingstoke, UK) medium for 24–48 h at 37 °C.

*H. pylori* ATCC 26695, gifted by Prof. Yong Xie from the First Affiliated Hospital of Nanchang University, was stored in brucella (Oxoid, UK) broth at −80 °C, incubated on Campylobacter Agar Base (CAB; Oxoid) supplemented with 5% defibrinated sheep blood and 1% mixed antibiotics (0.20 mg mL^−1^ polymyxin B, 0.30 mg mL^−1^ trimethoprim, 0.20 mg mL^−1^ amphotericin B and 0.25 mg mL^−1^ vancomycin; Yuanye, Shanghai, China) under microaerophilic conditions (5% O_2_, 10% CO_2_, and 85% N_2_) for 48 h at 37 °C. The liquid medium used to culture *H. pylori* was brain–heart infusion broth (BHI; Oxoid) with 10% fetal bovine serum (FBS; Gibco, Waltham, MA, USA).

### 2.2. Animal Experimental Design

The animal assays were performed according to the guidelines for the Ethical Review of Laboratory Animal Welfare of China National Standard [27] and approved by the Laboratory Animal Welfare Ethics Committee of Nanchang University (Project No. SYXK (Gan) 2022-0001). A total of 74 male C57BL/6 mice aged 4–6 weeks (20–24 g) were purchased from Vitality River, Beijing, and were divided into two large groups, except for the control group (8 mice), to perform the therapy (42 mice) and prophylaxis (24 mice) tests of *Lactobacillus* against *H. pylori* infection in mice. The schematic diagram is shown in Figure 1. In the therapy trials, HP-infected mice models were first constructed. All mice (eight per group), except the control group, received *H. pylori* solution (10^8^ CFU mL^−1^, 20 μL g^−1^) by gavage once every other day for four weeks. One week after infection, the mice (*n* = 2) were sacrificed and tested for infection. Then, HP-infected C57BL/6 mice were randomly divided into five groups—HP_NaCl, HP_LP61, HP_LS03, HP_Ant, and Ant_LS03 – for multimodal therapy. Details of the corresponding intragastric administration are given below:

- Control group: 0.9% NaCl gavage (9 weeks)
- HP_NaCl group: HP (4 weeks) + 0.9% NaCl (4 weeks)
- HP_LP61 group: HP (4 weeks) + *L. plantarum* CICC 20261 (10^8^ CFU mL^−1^, 4 weeks)
- HP_LS03 group: HP (4 weeks) + *L. salivarius* NCUH062003 (10^8^ CFU mL^−1^, 4 weeks)
- HP_Ant group: HP (4 weeks) + mixed antibiotics (0.025 μg mL^−1^ omeprazole, 0.125 μg mL^−1^ amoxicillin and 0.5 μg mL^−1^ metronidazole, 14 days)
- Ant_LS03 group: HP (4 weeks) + mixed antibiotics (14 days) with *L. salivarius* NCUH062003 (10^8^ CFU mL^−1^, 4 weeks).

In the prophylactic trial, the mice were randomly assigned to three groups: NaCl_HP, LP61_HP, and LS03_HP. Mice in each group initially received gavage with sterile saline or *Lactobacillus* for 4 weeks. Then, mice were administered *H. pylori* 26695 (10^8^ CFU mL^−1^) every other day for 4 weeks, except for the control group. Details of the corresponding intragastric administration are given below:

- Control group: 0.9% NaCl gavage (9 weeks)
- NaCl_HP group: 0.9% NaCl (4 weeks) + HP (4 weeks)
- LP61_HP group: *L. plantarum* CICC 20261 (10^8^ CFU mL^−1^, 4 weeks) + HP (4 weeks)
- LS03_HP group: *L. salivarius* NCUH062003 (10^8^ CFU mL^−1^, 4 weeks) + HP (4 weeks)

After stopping administration, mice were sacrificed by cervical dislocation in the tenth week. Serum samples were collected from retro-orbital blood by centrifugation (2000 g, 10 min) and stored at −80 °C. The stomachs of mice were excised, dissected along with the greater and lesser curvature, and rinsed using sterile PBS (0.01 M, pH 7.2–7.4), and the gastric body and antrum were stored separately.

### 2.3. Organ Coefficients Determination

The body weights of all mice were recorded daily before sacrifice. On the sacrifice day, body and organ weights were weighed. Then, the coefficients of the heart, liver, spleen, lung, and kidney to body weight were calculated.

### 2.4. H. pylori Colony Count

A portion of gastric antrum tissue was taken for homogenization. Then, the homogenate was diluted, and 100 μL of the dilution was coated on CAB agar medium and incubated for 48 h. Finally, plate colony counting of *H. pylori* was performed.

### 2.5. Urease Test

Part of the gastric antrum tissue was homogenized to detect urease content by a modified phenol red method. In brief, the gastric tissue homogenates were incubated with the urea–phenol red solution (20% urea and 0.012% phenol, pH 6.5) for 1 h. The color change was observed, and the OD values were measured at 561 nm using a microplate reader (Thermo Fisher, Waltham, MA, USA).

### 2.6. Pathological Examination

Gastric tissue was fixed in 4% paraformaldehyde buffer (pH 7.4, Biosharp, Beijing, China) for 24 h. For histological analysis, paraffin embedding was performed, followed by micro-machine sectioning, xylene dewaxing, and hematoxylin and eosin (H&E) staining. Finally, the stained sections were observed under a light microscope. Representative images were randomly selected from several slides. To assess inflammation and mucosal damage, inflammatory cells in the lamina propria were counted on H&E-stained slides, and the entire mucosal layer was observed regionally. Inflammation was scored as follows: 1. fewer inflammatory cells, confined to the superficial mucosal layer; 2. denser inflammatory cells, more than 1/3 of the mucosal layer; 3. dense inflammatory cells, occupying the whole mucosal layer; 4. few neutrophils infiltrated in the lamina propria; 5. more neutrophils infiltrated in the lamina propria; 6. denser neutrophils in the lamina propria and visible small concave abscesses. Mucosal damage was scored as follows: 1. normal mucosa; 2. damaged epithelial cells; 3. damaged glandular cells; 4. vesicles, hemorrhages, and ulcers in the mucosal layer.

### 2.7. Immunological Factor Analysis

The levels of serum IL-1β, IL-6, and IL-10 were determined using an ELISA Kit (Saipei, Wuhan, China). The serum frozen at −80 °C was thawed to room temperature. Diluted serum was incubated with the ELISA assay solution in a microplate reader according to the manufacturer’s instructions, and the OD value was measured immediately at 450 nm.

### 2.8. MPO and SOD Analyses

Myeloperoxidase (MPO) and superoxide dismutase (SOD) activities were quantified in gastric tissues. Firstly, the gastric tissue was homogenized with PBS. Then, the cell supernatant was collected by centrifugation (4000× *g*, 10 min) to determine MPO and SOD, respectively, via commercial kits (Saipei, Wuhan, China). The absorbance values were measured using a microplate reader at 450 nm.

### 2.9. Immunohistochemistry (IHC) Analysis

The sections of embedded fixed gastric tissue samples obtained using the slicing machine were processed for immunohistochemistry analysis (IL-1β and TGF-β) to evaluate inflammation. HRP-labeled goat anti-rabbit IgG was used as the secondary antibody. Color development for immunohistochemical staining was carried out using DAB (3, 3′-Diaminobenzidine) chromogenic solution, and hematoxylin was used to re-stain nuclei. The results were interpreted under a white-light microscope.

### 2.10. Immunofluorescence (IF) Assay

Immunofluorescent heterologous double-labeled ki67 and β-catenin staining by paraffin sections was used to assess apoptosis in gastric epithelial cells. Alexa Fluor 488-labeled goat anti-rabbit IgG and CY3-labeled goat anti-mouse IgG were used as secondary antibodies. Further, immunofluorescent homologous double-labeled ZO1 and Occludin staining by paraffin sections was used to assess the repair of the gastric mucosa. HRP-labeled goat anti-rabbit IgG and CY3-labeled goat anti-rabbit IgG were used as secondary antibodies. Double immunofluorescence staining was achieved using TSA (Tyramide signal amplification), followed by re-staining of nuclei using DAPI (4′,6-diamidino-2-phenylindole). Finally, the images were observed and captured in a fluorescence microscope at the corresponding wavelengths.

### 2.11. Gut Microbe 16S rRNA Sequencing and Data Analysis

The microbial genomic DNA was extracted from fecal samples with a MagBeads FastDNA Kit (MP Biomedicals, Irvine, CA, USA). The DNA quantity and quality were determined using the NanoDrop Spectrophotometer 2000 (Thermo Fisher, USA) and 1.2% agarose gel electrophoresis. The V3-V4 regions of the 16S rRNA gene were amplified by using the forward primer (338F 5′-ACTCCTACGGGAGGCAGCA-3′) and the reverse primer (806R 5′-GGACTACHVGGGTWTCTAAT-3′) [28]. Then, the DNA libraries were constructed using bacterial amplicons based on index and barcode information. Genome sequencing was performed on the Illumina Novaseq platform. Sequence analysis was performed by calling the Divisive Amplicon Denoising Algorithm 2 (DADA2) on the QIIME 2 platform (https://qiime2.org/ accessed on 19 September 2024) to denoise and dereplicate the data. The variants in the dereplicated sequence produced using DADA2 v1.14 quality control are called amplicon sequence variants (ASVs). All downstream data analyses were performed using the QIIME 2 and R v4.2.2 package. Alpha diversity metrics (Chao1, observed species, Shannon and Simpson indices) were calculated using the ‘qiime diversity alpha-rarefaction’ command from QIIME 2. Beta diversity metrics were calculated based on Bray–Curtis’s distance matrix between samples [29] and visualized via principal coordinate analysis (PCoA) [30] and hierarchical clustering analysis (HCA) [31]. An analysis of similarities (Anosim) non-parametric test was used to distinguish between-group variation and to calculate the test statistic’s R-value [32,33]. Linear discriminant analysis effect size (LEfSe) was used to detect taxa rich in between-group differences at the phylum and genus levels [34]. PICRUSt2 V2.5.1 software was used to predict the functional abundance based on the abundance of marker gene sequences in the samples to obtain functional units and, based on metabolic pathway databases and certain calculation methods, to obtain the abundance values of metabolic pathways for metabolic pathway statistics [35]. Finally, the results of species functional differences were analyzed using STAMP v2.1.3 software [36].

### 2.12. Fecal Short-Chain Fatty Acid Determination

Feces were suspended in ultrapure water at 1:4 (*w*:*w*), and sonicated for 5 min at 4 °C. The mixture was adjusted to pH 2.0–3.0 with HCl solution (5 mol L^−1^). After centrifugation (8000× *g*, 20 min, 4 °C), the supernatant was obtained and filtered through a 0.22 μm membrane. 2-Ethyl butyric acid was added to the supernatant (1 mmol L^−1^) as an internal standard. The supernatant (1 μL) was ionized by a GC-QTOF/MS ion source (Agilent, Santa Clara, CA, USA) and detected by an autosampler, and the short-chain fatty acids (SCFAs) were separated in a DB-WAX gas chromatographic column (Agilent, USA). The heating procedure was as follows: keep the starting temperature at 60 °C for 2.5 min, increase the temperature to 150 °C at 10 °C min^−1^ for 5 min, and increase the temperature to 250 °C at 10 °C min^−1^ for 2 min. For the mass spectrometry parameters, the sample was injected by a shunt (shunt ratio of 1:20), the carrier gas was helium (flow rate of 1.0 mL min^−1^), and the injector and detector temperatures were set at 250 °C and 260 °C with an energy of 70 eV. Finally, compound identification was performed by matching the collected mass spectrometry data with the NIST database [37], and compound semi-quantitative analyses were performed using the ratio of 2-Ethyl butyric acid concentration to peak area [38].

### 2.13. Statistical Analysis

Statistical analyses were performed using SPSS Statistics 27 and differences between groups were compared using ANOVA one-way analysis of variance. Data were mean ± standard deviation (*n* = 8). Graphs were plotted using Origin 2024b. Different lowercase letters in the bar graphs indicated significant differences (*p* < 0.05).

## 3. Results and Discussion

### 3.1. Body Weight and Organ Coefficient

As shown in Table 1 and Figure S1B, within the 70-day experimental period, the body weights of all mice increased normally with age, and there was no significant difference in the body weights of all groups of mice. In calculating the organ coefficients of the heart, liver, spleen, lungs, and kidneys, the liver coefficients of the mice in the HP_Ant group were found to be significantly increased, suggesting that the livers were edematous, congested, or hypertrophied. This indicates that the gavage of amoxicillin/metronidazole increased the burden on the liver of mice, which had already been damaged during the metabolism of antibiotics [39]. Meanwhile, the liver coefficients of mice in the Ant_LS03 group were at normal levels, indicating that *L. salivarius* NCUH062003 helps to mitigate antibiotic-induced liver damage in mice. Finally, the heart, liver, spleen, lungs, and kidneys of the mice in each group were examined by H&E staining, as shown in Figure S1A, and no obvious pathological changes were found in the HP_LS03 and LS03_HP groups, suggesting that *L. salivarius* NCUH062003 has good compatibility in vivo.

### 3.2. Effects of L. salivarius NCUH062003 on H. pylori Activity in Gastric Antrum of HP-Infected Mice

**Figure 2:**
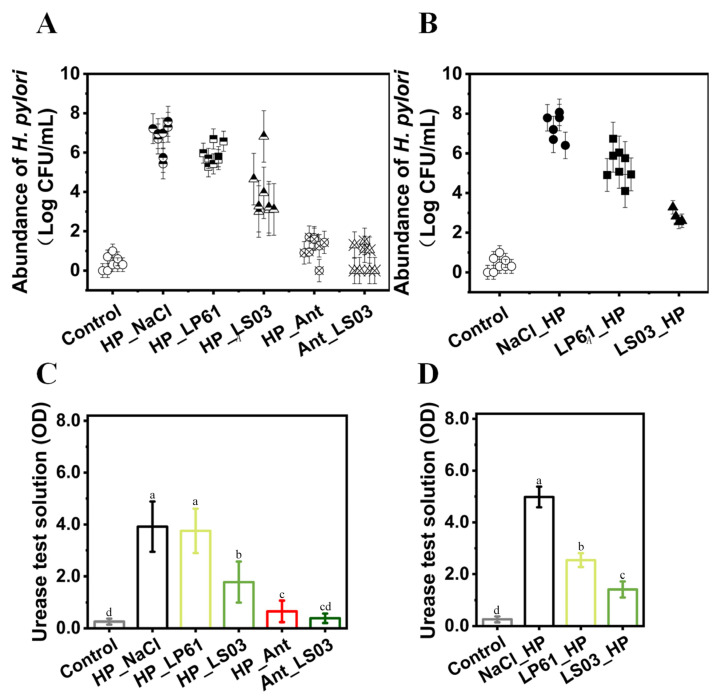
(**A**,**B**) Determination of the abundance of *H. pylori* in the gastric tissues of mice in therapeutic and prophylactic groups. (**C**,**D**) Determination of the urease activity of the mouse gastric mucosa in therapeutic and prophylactic groups. Therapeutic groups: (1) control, (2) HP_NaCl, (3) HP_LP61, (4) HP_LS03, (5) HP_Ant, (6) and Ant_LS03 groups. Prophylactic groups: (1) control, (7) NaCl_HP, (8) LP61_HP, (9) and LS03_HP groups. LP61: *L. plantarum* CMCC 20261. LS03: *L. salivarius* NCUH062003 ANT: 0.125 μg mL^−1^ amoxicillin and 0.5 μg mL^−1^ metronidazole. A total of 9 groups were formed, with 8 mice in each group. Different lowercase letters in the bar graphs indicated significant differences (*p* < 0.05).

The in vivo efficacy of *L. salivarius* NCUH062003 for the therapy of *H. pylori* infection was first evaluated. After 4 weeks of *H. pylori* gavage, four mice were randomly selected for dissection, and the gastric tissues were subjected to plate coating, hematoxylin–eosin (H&E) staining, and a urease assay, which indicated successful modeling of HP-infected mice (Figure S2). Next, the mice were subjected to intragastric administration of *Lactobacillus* or drug therapy. Then, the mice were sacrificed, and gastric tissues were taken in the 10th week for biochemical experiments. The results of plate counting show that the HP_NaCl group contained the largest *H. pylori* colonization rate at 6.74 Lg CFU mL^−1^, and the HP_LP61, HP_LS03, HP_Ant, and Ant_LS03 groups had *H. pylori* colonization rates of 5.88, 3.89, 1.19, and 0.61 Lg CFU mL^−1^, respectively (Figure 2A). Also, the urease absorbances in the HP_NaCl and HP_LP61 groups were 3.91 ± 0.97 and 3.75 ± 0.86, respectively, whereas the urease absorbances in the HP_LS03, HP_Ant, and Ant_LS03 groups were 1.78 ± 0.79, 0.66 ± 0.42, and 0.39 ± 0.18, respectively (Figure 2C and Table S1). In addition, within the prophylactic group, in the NaCl_HP, LP61_HP, and LS03_HP groups, the *H. pylori* colonization rates were 7.17, 5.97, and 3.22 Lg CFU mL^−1^ (Figure 2B), and the urease absorbances were 4.59 ± 0.66, 2.84 ± 0.35, and 1.44 ± 0.26, respectively (Figure 2D and Table S1). Taken together, these findings indicate that *L. salivarius* NCUH062003 reduced *H. pylori* colonization in the gastric mucosa, and the combination of *L. salivarius* NCUH062003 and antibiotics reduced *H. pylori* colonization even more significantly, and, more importantly, *L. salivarius NCUH062003* was more effective at preventing *H. pylori* colonization in the gastric mucosa of mice.

Our previous findings showed that the supernatant and live cells of *L. salivarius* NCUH062003 reduced *H. pylori* urease activity by 70.94% and 83.67%, respectively, in vitro, and antagonized *H. pylori* by inducing coccoid conversion and intercellular adhesion [26], so its supernatant and cells collectively formed a chemical and biological membrane barrier that enhanced the host’s gastric mucosal barrier. The antibacterial metabolites of the NCUH062003 supernatant include lactic acid, which is predominant, as well as hydrogen peroxide and bacteriocins. In addition, *L. salivarius* NCUH062003 had high adhesion to gastric mucin and gastric adenocarcinoma cells at 19.25% and 11.83%, respectively. Studies have shown that probiotics have an ‘occupancy effect’ in the gastric mucosa, which can prevent the adhesion of pathogenic bacteria [40], and the exclusion effect of *L. salivarius* NCUH062003 on *H. pylori* adherence to gastric mucin and AGS cells was better than the replacement effect [26]. So, when *L. salivarius* NCUH062003 occupies more sites, *H. pylori* will compete and be excluded. The occupancy effect of *L. salivarius* NCUH062003 could explain its intervention in HP-infected mice colonized in vivo in this study, where the preventive effect was better than the therapy effect. Meanwhile, the genome of *L. salivarius* NCUH062003 was found to contain a secondary metabolite biosynthesis gene cluster, T3PKS, which contained 4 ribosomal natural product synthesis genes out of 49 genes, encoding one thiopeptide and three lanthipeptide, respectively. Moreover, a heat-stable bacteriocin (4.1 kDa–6.5 kDa) was purified from the supernatant, which has an efficient antagonistic ability against *H. pylori* [26].

### 3.3. Effects of L. salivarius NCUH062003 on Gastric Histopathology in HP-Infected Mice

**Figure 3:**
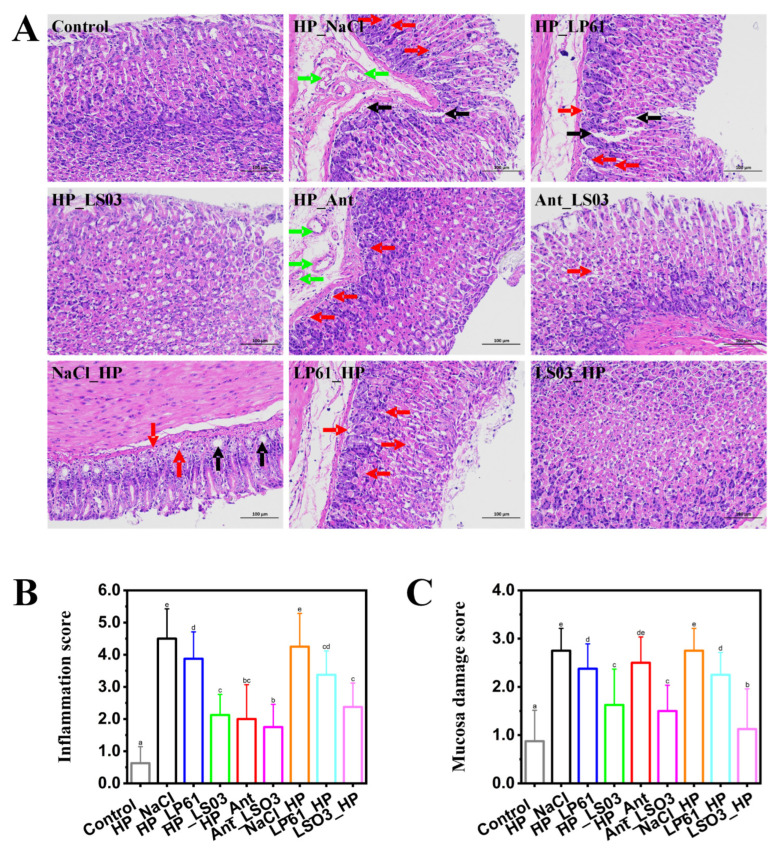
(**A**) Histopathology of gastric antrum tissue in the pyloric part of mice from different groups as determined by hematoxylin and eosin staining (200×). (**B**) Gastric mucosal lymphocyte infiltration score. (**C**) Gastric mucosal injury score. Therapeutic groups: control, HP_NaCl, HP_LP61, HP_LS03, HP_Ant, and Ant_LS03 groups. Prophylactic groups: control, NaCl_HP, LP61_HP, and LS03_HP groups. Black arrow: massive vacuole formation in lamina propria of gastric mucosa. Red arrow: neutrophil and lymphocyte infiltration in epithelial layer and lamina propria; green arrow: erythrocytes and hemorrhage in muscularis mucosa. Different lowercase letters in the bar graphs indicated significant differences (*p* < 0.05).

The pathological results of H&E-staining gastric antrum tissues in different groups of mice are shown in Figure 3A and Table S2. The gastric mucosa of the control group was clear in structure, with normal morphology, an intact epithelium, and tightly arranged mucosal glands. In the therapy (HP_NaCl) and prophylactic group (NaCl_HP) *H. pylori*-infected model mice, the epithelial cells and some glandular cells of the gastric mucosa were damaged, with many vacuoles’ formation (black arrowheads) and neutrophils infiltrating the epithelial and lamina propria (red arrowheads). In the therapy trial, the gastric mucosal structure in the LS03_HP group tended to be clear and intact and relatively tightly arranged compared with the NaCl_HP and LP61_HP groups. In contrast, some erythrocytes (green arrows) appeared in the muscularis mucosae of the antibiotic therapy group (HP_Ant), which showed bleeding and ulceration; the tissue damage of the gastric mucosa was recovered after 4 weeks of therapy with *L. salivarius* NCUH062003 combined with antibiotic therapy (Ant_LS03). In addition, in the prophylactic trial, slight lymphocytic infiltration (red arrows) was still present in the gastric histopathological sections of the HP_LS03 group, but there was a significant reduction in neutrophils in the lamina propria, disappearance of neutrophils in the lamina propria, and hemorrhage in the mucosal muscularis layer.

The gastric mucosa is the innermost layer of the stomach and consists of the epithelium, lamina propria, and mucosal muscularis layer, comprising three protective mucosal barriers [41]. Histological changes in the gastric mucosa are one of the most important and obvious manifestations of *H. pylori* infection [42]. The eradication efficiency of *H. pylori* can be assessed with inflammatory indicators (elevated lymphocytes) and histological activity indicators (neutrophil polymorphic density) [43]. In addition, some studies have reported that neutrophils disappear at an early stage after *H. pylori* eradication; therefore, inflammation and activity scores are considered highly sensitive indicators for assessing the presence or absence of *H. pylori* [44]. The gastric mucosal lymphocyte infiltration score (Figure 3B) and gastric mucosal damage score (Figure 3C) indicated that *L. salivarius* NCUH062003 intragastric administration ameliorates gastric mucosal damage induced by antibiotic therapy in mice and can be used as a prophylactic and adjunctive therapy for inflammatory responses caused by *H. pylori* infection.

### 3.4. Alleviation of L. salivarius NCUH062003 on Gastric Mucosal Inflammation in HP-Infected Mice

**Figure 4:**
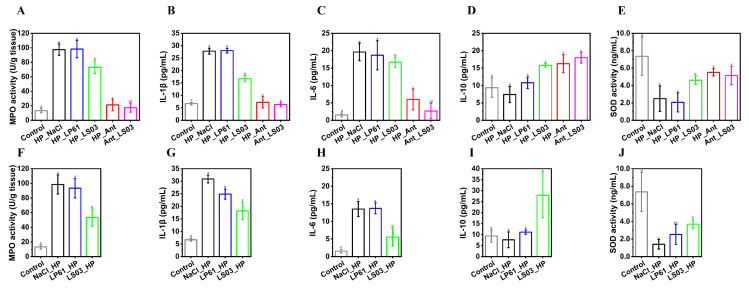
Determination of MPO activity (**A**,**F**) and SOD levels (**E**,**J**) in gastric tissue of mice and the levels of pro-inflammatory factors IL-1β (**B**,**G**) and IL-6 (**C**,**H**) and anti-inflammatory factor IL-10 (**D**,**I**) in the serum of mice in the prophylactic and therapeutic groups, as determined by enzyme-linked immunosorbent assay (ELISA). Therapeutic groups: control, HP_NaCl, HP_LP61, HP_LS03, HP_Ant, and Ant_LS03 groups. Prophylactic groups: control, NaCl_HP, LP61_HP, and LS03_HP groups. Different lowercase letters in the bar graphs indicated significant differences (*p* < 0.05).

**Figure 5:**
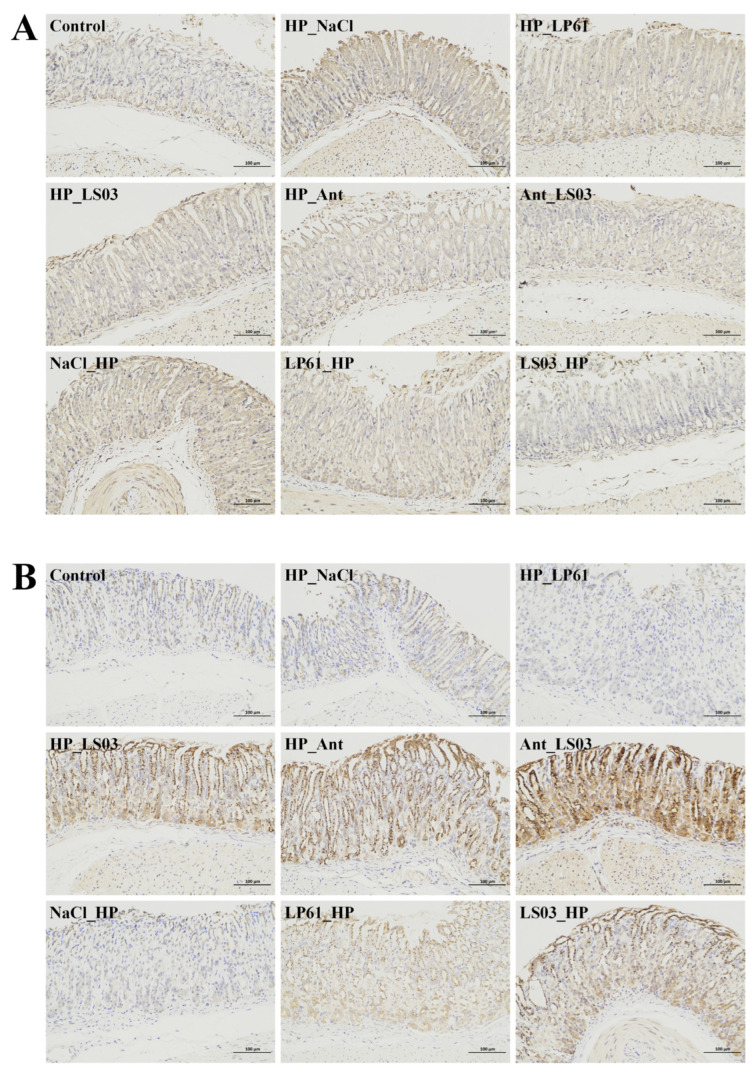
Immunohistochemical staining of pro-inflammatory IL-1β (**A**) and anti-inflammatory TGF-β (**B**) in mice gastric tissue in the therapeutic and prophylactic groups (200×). Hematoxylin-stained nuclei were blue, and DAB (3,3′-Diaminobenzidine) showed positive expression in a brownish color. Therapeutic groups: control, HP_NaCl, HP_LP61, HP_LS03, HP_Ant, and Ant_LS03 groups. Prophylactic groups: control, NaCl_HP, LP61_HP, and LS03_HP groups.

As shown in Figure 4A,F, myeloperoxidase (MPO) levels in gastric mucosal tissues of mice in the HP_NaCl and NaCl_HP groups were higher than those in other groups, whereas MPO levels were significantly reduced in the HP_LS03, Ant_LS03, and LS03_HP groups. In addition, ELISA and immunohistochemistry results show that the *L. salivarius* NCUH062003 intervention also effectively reduced the levels of the pro-inflammatory cytokines interleukins IL-1β and IL-6 while increasing the levels of anti-inflammatory factors IL-10 and TGF-β (Figure 4B–D,G–I and Figure 5). Meanwhile, *L. gasseri* LG21, an excellent anti-*H. pylori* strain screened by Japan’s Meiji Dairy, was only able to inhibit the pro-inflammatory cytokine factor IL-8 induced by *H. pylori* [45].

Myeloperoxidase (MPO) activity is an indicator of the extent of neutrophil infiltration, and MPO catalyzes the synthesis of a variety of cytotoxic oxidants, which are further exacerbated by increased gastric mucosal damage [46]. After *H. pylori* colonizes the host, its self-expressed urease, neutrophil-activating protein, and other proteins act as antigens and mediate neutrophil chemotaxis [47]. Thereafter, neutrophils infiltrate the gastric mucosa and exert mucosal immunity by secreting cytokines and releasing molecules including MPO and reactive oxygen species [48]. Meanwhile, the H&E staining pathological results, gastric mucosal inflammation, and damage scores show that the intervention of *L. salivarius* NCUH062003 resulted in a significant decrease in neutrophils in the lamina propria and a reduction in hemorrhagic symptoms in the mucosal muscular layer of the gastric antrum in mice (Figure 3). The above results suggest that *L. salivarius* NCUH062003 can alleviate the inflammatory response of the gastric mucosa by inhibiting *H. pylori*-mediated neutrophil chemotaxis.

### 3.5. Restoration of L. salivarius NCUH062003 on Gastric Mucosa of HP-Infected Mice

**Figure 6:**
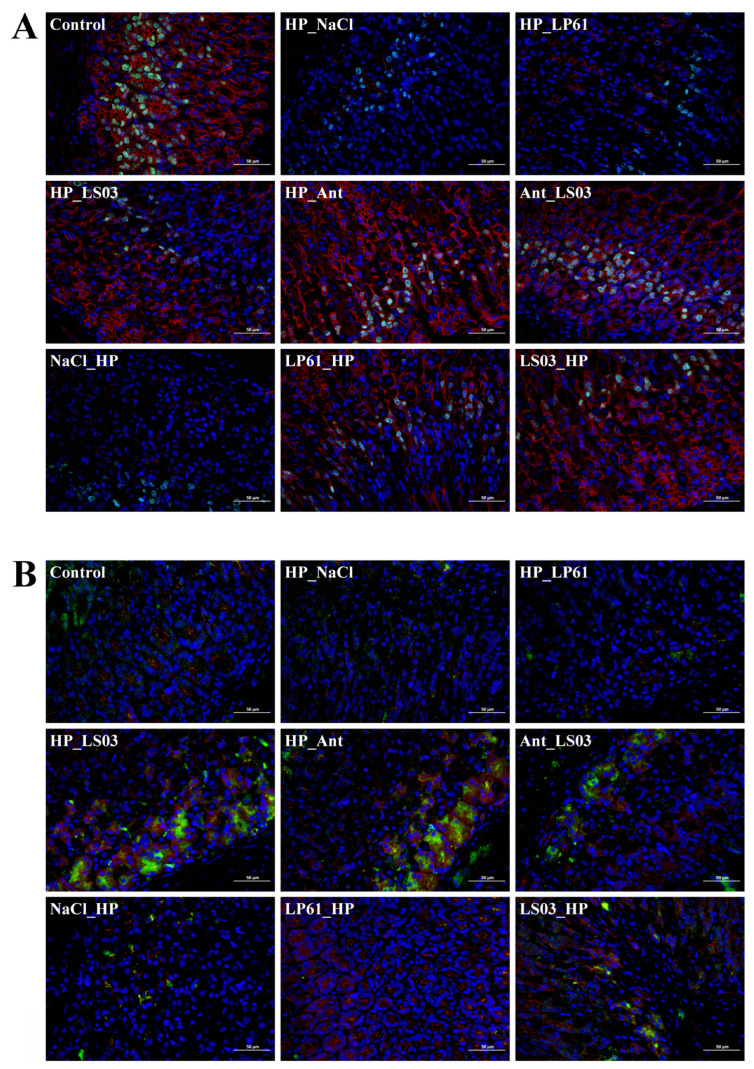
(**A**) Immunofluorescence heterologous double-labeled Ki-67 and β-catenin staining images in gastric tissues used to assess the apoptosis of gastric epithelial cells. (**B**) Immunofluorescence homologous double-labeled staining images of Occludin and ZO1 proteins involved in gastric mucosal epithelial repair (400×). DAPI channel nuclei appear blue, 488 channel positivity appears green, and CY3 channel positivity appears red. Therapeutic groups: control, HP_NaCl, HP_LP61, HP_LS03, HP_Ant, and Ant_LS03 groups. Prophylactic groups: control, NaCl_HP, LP61_HP, and LS03_HP groups.

Inflammation-induced oxidative stress is mediated by reactive oxygen species, which induce changes in mitochondrial membrane potential and permeability [49]. Also, reactive oxygen species not only translocate apoptosis-associated proteins in the mitochondrial membrane but also release apoptosis-associated factors into the cytoplasm, which leads to apoptosis in the mitochondrial pathway [50]. Ki-67 is a nuclear antigen specifically associated with cell proliferation [51], whereas β-catenin is an important participant in the classical Wnt signaling pathway for cell proliferation and differentiation and is also a core protein molecule in the tyrosine-protein kinase signal transduction pathway [52]. Therefore, in this study, immunofluorescence was used to detect the expression of Ki-67 and β-catenin in the gastric mucosa of mice. Immunofluorescence staining images of mice gastric tissues showed that the number of gastric epithelial cells in the HP_LS03, Ant_LS03, and LS03_HP groups was close to that of the control group, suggesting that *L. salivarius* NCUH062003 has a good ability to resist apoptosis (Figure 6A). Furthermore, ZO1 tight junction protein, a scaffolding protein, attaches tight junction transmembrane proteins to the actin cytoskeleton [53], and Occludin, a membrane-integrating protein, is expressed at tight junctions of epithelial and endothelial cells [54]. ZO -1 protein interacts with Occludin protein, forming a complex that mediates intercellular tight junctions and signal transduction, which regulates paracellular permeability and maintains cell polarity [55]. Therefore, immunofluorescence staining showed that ZO1 and Occludin protein expression was up-regulated in the gastric tissues of mice in the HP_LS03, HP_Ant, Ant_LS03, and LS03_HP groups, and the protein levels tended to normalize (Figure 6B).

Moreover, as shown in Figure 4E,G, *L. salivarius* NCUH062003 intervention increased the level of superoxide dismutase (SOD) in the gastric mucosal tissue of mice, suggesting that *L. salivarius* NCUH062003 possesses an antioxidant stress effect. SOD is an important antioxidant that protects cells from oxidative damage by scavenging free radical-induced intracellular production of superoxide anion radicals [56]. And some research has shown that exogenous SOD can effectively attenuate acute gastric mucosal injury induced by different factors [57]. Taken together, these findings indicate that *L. salivarius* NCUH062003 can restore *H. pylori*-injured gastric mucosa by resisting gastric epithelial cell apoptosis and promoting the expression of gastric mucosal repair proteins in mice gastric tissues.

### 3.6. Effect of Therapy Modalities on the Gut Microbiota of HP-Infected Mice

#### 3.6.1. Diversity of Gut Microbiota

**Figure 7:**
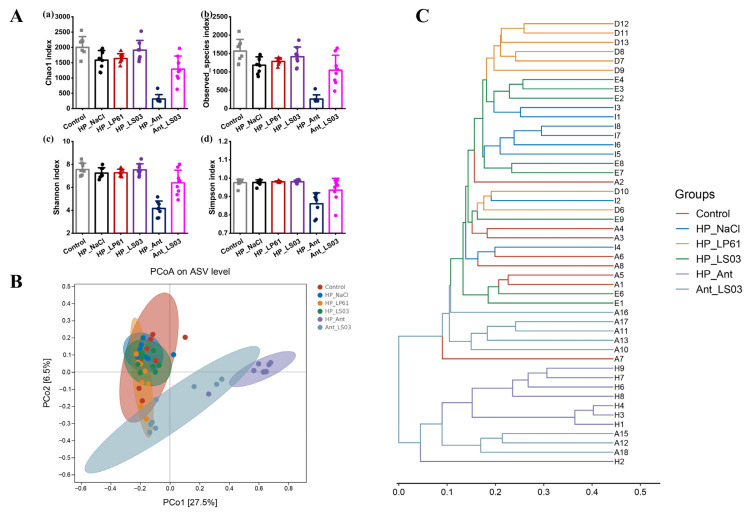
(**A**) Alpha diversity index of the mice gut microbiota samples in each therapeutic group: (**a**) ACE index, (**b**) Chao1 index, (**c**) Shannon index, and (**d**) Simpson index. (**B**) The PCoA chart of the gut microbiota of mice in each therapeutic group. (**C**) The hierarchical clustering tree diagram of the gut microbiota of mice in therapeutic groups. Therapeutic groups: control, HP_NaCl, HP_LP61, HP_LS03, HP_Ant, and Ant_LS03 groups. A total of 6 groups were formed, with 8 mice in each group.

Alpha diversity and beta diversity indices can characterize species diversity within and between habitats, respectively, to provide a comprehensive evaluation of community diversity [58,59]. Alph diversity includes the Chao1 index [60] and observed species index [61] characterizing species richness and the Shannon index [62] and Simpson index [63] characterizing species diversity. The effect of *Lactobacillus* or antibiotics in therapy mode on the alpha diversity of the gut microbiota of HP-infected mice is shown in Figure 7A. The Chao1 index and observed species index in the HP_NaCl group were significantly lower than those in the control group (*p* < 0.01), the Shannon index was slightly lower than that in the control group (*p* < 0.05), and there was no significant change in the Simpson index (*p* > 0.05), which indicated that *H. pylori* significantly reduced the species richness of the mice gut microbiota and had a certain degree of negative impact on the species diversity of the mice gut microbiota. *L. plantarum* LP61 and *L. salivarius* NCUH062003 mitigated this negative effect to varying degrees after their respective interventions, with *L. salivarius* NCUH062003 mitigating it to a greater extent. In addition, the Chao1 index, observed species index, and Shannon index in the LS03 group were significantly higher than those in the HP_NaCl group (*p* < 0.01) and showed no significant change from the control group (*p* > 0.05), and the Simpson index in the LS03 group was not significantly different from that in HP_NaCl or the control groups (*p* > 0.05), whereas the Chao1 index and observed species index of the LP61 group were significantly higher than those of the HP_NaCl group (*p* < 0.05) but significantly lower than those of the LS03 group (*p* < 0.05). The Shannon index of the LP61 group was not significantly different from that of the HP_NaCl group (*p* > 0.05) and was significantly lower than that of the control group (*p* < 0.05), and the Simpson’s index in the LP61 group was not significantly different (*p* > 0.05) compared to the HP_NaCl and control groups. These results indicate that *L. salivarius* NCUH062003 restores alpha diversity by significantly alleviating the species richness and species diversity of the gut microbiota of *H. pylori*-affected mice, whereas the control strain, *L. plantarum* LP61, partially restores the species richness of the gut microbiota of HP-infected mice but does not have a significant effect on the species diversity of the gut microbiota. Furthermore, the Chao1 index, observed species index, Shannon index, and Simpson index were all highly significantly lower in the Ant group than in the control group (*p* < 0.001) and significantly lower than in the HP_NaCl group (*p* < 0.01), suggesting that amoxicillin/metronidazole significantly reduced mice gut microbiota species richness and species diversity, and the negative effect of amoxicillin/metronidazole on the diversity of the mice gut microbiota was significantly higher than that caused by *H. pylori*. This negative effect was significantly mitigated after a four-week intervention with *L. salivarius* NCUH062003 performed concurrently with and at the end of the antibiotic therapy. The Chao1 index, observed species index, Shannon index, and Simpson index were significantly higher in the Ant_LS03 group than in the HP_NaCl group (*p* < 0.01), but significantly lower than in the LS03 and control groups (*p* < 0.01). These results suggest that *L. salivarius* NCUH062003 significantly attenuates the negative effects of amoxicillin/metronidazole on species richness and species diversity of the mice gut microbiota, thereby partially restoring alpha diversity.

Beta diversity is the dissimilarity of species composition between habitats [64], downscaling multidimensional microbial data and demonstrating data trends through principal coordinates analysis (PCoA) [29], and identifying discontinuous object subsets and classifying data through clustering analysis [65]. The effect of Lactobacillus or antibiotics in therapy mode on the beta diversity of the gut microbiota of HP-infected mice is shown in Figure 7B and Table S3. The gut microbiota in each group were clustered individually, with good intra-group aggregation and inter-group separation. The PCoA plot showed a large sample difference distance between the HP_NaCl and control groups of the mice gut microbiota (R = 0.390, *p* = 0.002), with the HP_LP61 group (R = 0.352, *p* = 0.001) and the HP_LS03 group (R = 0.272, *p* = 0.001) between the HP_NaCl and control groups, with the HP_LS03 group closest to the control group. The results show that *H. pylori* significantly altered the beta diversity of the mice gut microbiota, and *L. salivarius* NCUH062003 alleviated the H. pylori-induced beta diversity distance. In addition, the PCoA plot [30] showed a greater sample difference distance for the mice gut microbiota in the HP_Ant group than in the control group (R = 0.971, *p* = 0.001), whereas the sample difference distance for the gut microbiota of mice in the Ant_LS03 was significantly shorter than that in the control group (R = 0.436, *p* = 0.008). The results show that amoxicillin/metronidazole significantly altered the beta diversity of the gut microbiota of HP-infected mice and was the largest contributor to species composition heterogeneity expansion of the mice gut microbiota. *L. salivarius* NCUH062003 significantly reduced the beta diversity distance due to amoxicillin/metronidazole therapy, thereby narrowing the mice gut microbiota heterogeneity. Furthermore, hierarchical clustering is the presentation of similarity between samples in the form of a hierarchical tree to determine the intermittency of the data [31]. The hierarchical cluster analysis of the mice gut microbiota in each group in the therapy model is shown in Figure 7C. All samples from the control, HP_NaCl, HP_LP61, and HP_LS03 groups were clustered with samples A10, A11, A13, A16, and A17 from the Ant_LS03 group, and all samples from the HP_Ant group were clustered with samples A12, A15, and A18 from the Ant_LS03 group. Samples from the Ant_LS03 group showed similarity both to samples from the HP_Ant group and to samples from the control, HP_NaCl, HP_LP61, and HP_LS03 groups, which were not treated with amoxicillin/metronidazole; although the gut microbiota species composition dissimilarity was largely restored in five samples from the Ant_LS03 group, significant discontinuities in the gut microbiota data of the other three samples remained. The results show that an additional four-week adjunctive intervention of *L. salivarius* NCUH062003 concomitant with two weeks of amoxicillin/metronidazole therapy did not completely change the differences in beta diversity caused by amoxicillin/metronidazole on the mice gut microbiota. Amoxicillin/metronidazole has a significant negative and prolonged effect on the mice gut microbiota and should prolong the duration of adjunctive intervention with *L. salivarius* NCUH062003.

#### 3.6.2. Composition of Gut Microbiota

**Figure 8:**
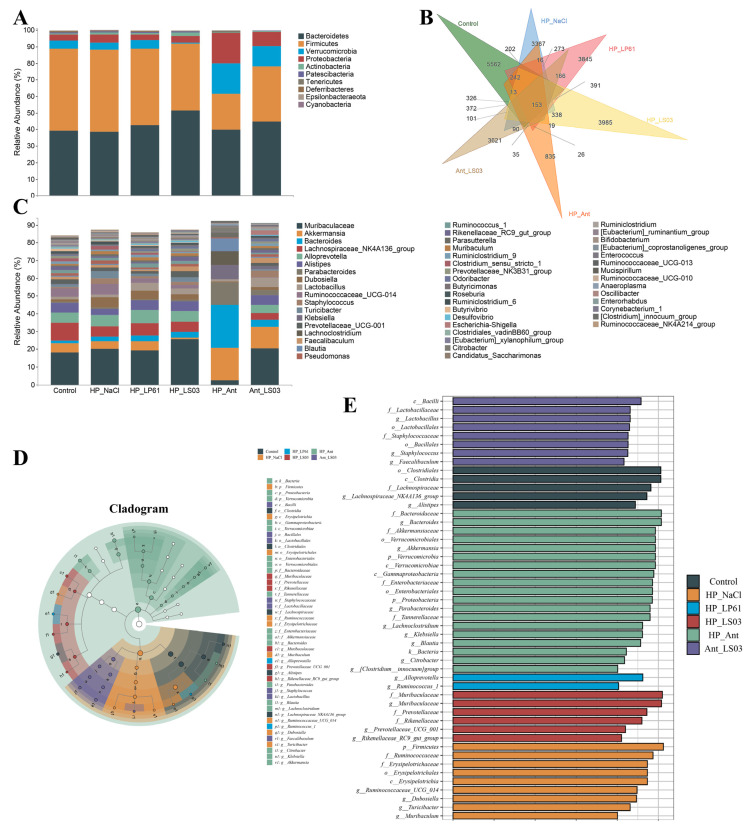
(**A**) The relative abundance of gut microbiota at the phylum level of mice in each therapeutic group. (**B**) Venn diagram of the gut microbiota of mice in each therapeutic group. (**C**) The relative abundance of gut microbiota at the genus level of mice in each therapeutic group. The taxonomic cladogram (**D**) and the histogram (**E**) from LEfSe analysis of the gut microbiota in therapeutic groups. Therapeutic groups: control, HP_NaCl, HP_LP61, HP_LS03, HP_Ant, and Ant_LS03 groups. A total of 6 groups were formed, with 8 mice in each group.

DiVenn plots were used to explore the number of shared and exclusive species of the mice gut microbiota in each therapy group [66]. As shown in Figure 8B, the number of exclusive ASVs of the gut microbiota of mice in the control group (5562) was much higher than that in the HP_NaCl group (3367), whereas the number of exclusive ASVs of the gut microbiota of mice in the LP61 group and the LS03 group was higher than that in the HP_NaCl group. The LS03 group had the highest number of exclusive ASVs (3985); meanwhile, the number of exclusive ASVs in the Ant group (835) was much lower than that in the Ant_LS03 group (3621). In addition, the highest number of shared ASVs with the control group was also in the LS03 group (1742), followed by the LP61 group (1635), and the HP_NaCl group (1544), while the lowest number of shared ASVs was found in the Ant group (242). The Ant_LS03 group (1311) shared a significantly higher number of ASVs with the control group than the Ant group. These results indicate that *H. pylori* reduced the number of exclusive species in the gut microbiota and that amoxicillin/metronidazole induced dysbiosis of the gut microbiota composition, and *L. salivarius* NCUH062003 and *L. plantarum* LP61 alleviated the reduction in the number of exclusive species in the gut microbiota induced by *H. pylori* to varying degrees. *L. salivarius* NCUH062003 restored the gut microbiota dysbiosis induced by amoxicillin/metronidazole with a better effect.

In addition, as shown in Figure 8A and Figure S3, the dominant phyla of the gut microbiota in all therapy groups were *Bacteroidetes* and *Firmicutes* (relative abundance 21.70–49.57% and 38.51–51.56%). Compared with in the control group, in the Ant group, the relative abundance of the *Bacteroidetes* and *Firmicutes* was significantly lower, and those of the *Verrucomicrobia* and *Proteobacteria* were significantly higher, whereas compared with in the Ant group, in the Ant_LS03 group, the relative abundance of *Bacteroidetes* and *Firmicutes* was significantly higher, while those of *Verrucomicrobia* and *Proteobacteria* were significantly lower. *Bacteroidetes* are highly successful competitors in the intestinal ecosystem with nutritional flexibility and the ability to respond to stresses exerted by the host [67]. Firmicutes is one of the dominant phyla of the gut microbiota, and many of these genera are beneficial bacteria that participate in a wide range of metabolic activities in the gut and can play a key role in the nutrition and metabolism of the host through short-chain fatty acid synthesis [68]. *Verrucomicrobia* is the fifth-largest gut microbiota [69], with a proportion of about 2% of the gut microbiota, and its abundance is closely related to human intestinal health, with some studies confirming that the proportion of *Verrucomicrobia* increases significantly after metabolic surgery [70]. *Proteobacteria*, in which all bacteria are Gram-negative, include many pathogenic organisms such as *Vibrio*, *Helicobacter*, *Escherichia*, *Campylobacter jejuni*, and *Yersinia coli*, which can cause serious diseases [71], and *Helicobacter pylori* also belongs to the *Proteobacteria* [72]. Thus, *L. salivarius* NCUH062003 intervention reversed the amoxicillin/metronidazole-induced decrease in *Bacteroidetes* and *Firmicutes* and the increase in *Verrucomicrobia* and *Proteobacteria*, suggesting that *L. salivarius* NCUH062003 has a restorative effect on the gut microbiota dysbiosis of HP-infected mice, and that this restorative effect correlates with its inhibition of the *Proteobacteria*, as well as with its alleviation of inflammation and intestinal damage.

Furthermore, at the genus level, the relative abundance of the mice gut microbiota in each therapy group is shown in Figure 8C and Figure S4. Compared to the control group, in the Ant group, the relative abundance of the *Akkermansia*, *Bacteroides*, *ParaBacteroides* and *Klebsiella* was highly significantly increased (*p* < 0.001), and the relative abundance of the *Muribaculacea*, *Lachnospiraceae*, *Alloprevotella*, *Alistipes*, *Dubosiella*, *Lactobacillus*, and *Faecalibaculum* was significantly decreased (*p* < 0.01). After *L. salivarius* NCUH062003 intervention, in the Ant_LS03 group, *Akkermansia*, *Bacteroides*, *ParaBacteroides* and *Klebsiella* were significantly lower than in the Ant group (*p* < 0.01) and the relative abundance of *Bacteroides*, *ParaBacteroides* and *Klebsiella* was decreased to a level that was not significantly different from that of the control group, whereas *Muribaculacea*, *Alloprevotella*, *Alistipes* and *Dubosiella* increased in relative abundance to a level that was not significantly different from that of the control group (*p* > 0.05), the relative abundance of *Lachnospiraceae* increased significantly but did not return to the level of the control group (*p* < 0.05), and the relative abundance of *Lactobacillus*, and *Faecalibaculum* both increased significantly and exceeded that of the control group (*p* < 0.01).

Even further, LEfSe analysis was used to explore the key differential microorganisms in the gut microbiota of mice in each therapy group [34]. As shown in Figure 8D,E, at the genus level, *Clostridia* and *Lachnospiraceae* were significantly enriched in the control group; *Ruminococcaceae* and *Erysipelothrix* were significantly enriched in the HP_NaCl group; *Alloprevotella* and *Ruminococcaceae* were significantly enriched in the HP_LP61 group; *Muribaculaceae*, *Prevotellaceae,* and *Rikenellaceae* were significantly enriched in the HP_LS03 group; *Akkermansia*, *Klebsiella*, *Citrobacter*, and *Clostridium* were significantly enriched in the Ant group; and *Lactobacillus* and *Faecalibaculum* were significantly enriched in the Ant_LS03 group. *Erysipelothrix* is a podless, non-budding, non-flagellated Gram-positive bacteria that causes swine dengue and other infections in animals and humans [73]. Although *Bacteroides* and *ParaBacteroides* are important cornerstone genera of the intestinal tract, some genera may become opportunistic pathogens in the context of GI trauma, cancer, or GI surgery, e.g., *Bacteroides fragilis* can invade the submucosal layer and cause purulent infections in body organs, such as the intestine, the abdominal cavity, and brain tissues with abscesses, which also contributes to the development of colon and rectal cancer [74]. *Akkermansia*, a genus in the phylum *Verrucomicrobia*, is not independently pathogenic, but its progression from adhesion to degradation of the intestinal mucus layer involves initial pathogenic behavior and can facilitate *Salmonella typhimurium* invasion of the host, leading to exacerbation of the intestinal inflammation [75]. *Klebsiella* belongs to the family of Enterobacteriaceae and is extremely pathogenic, being one of the most important conditionally pathogenic and medically infectious organisms, which can cause disease in humans and a wide range of animals [76]. *Citrobacter* is a conditionally pathogenic bacterium that causes diarrhea and extra-intestinal infections such as septicemia, meningitis, and brain abscesses, thereby adversely affecting the health and even the life of the host [77]. *Clostridia* is a member of the gut microbiota that can produce spores that produce a variety of toxins that involve various parts of the body, such as muscles, the digestive tract, and tissues [78]. Intestinal core genera are important components of the human body to maintain health and stability, and when most core genera are at a disadvantage with low abundance, harmful intestinal bacteria gradually begin to prevail [79]. However, *Lachnospiraceae* [80], *Alloprevotella* [81], *Alistipes* [82], *Dubosiella* [83], and *Lactobacillus* [84] are producers of short-chain fatty acids (SCFAs), which are important for the provision of energy, maintenance of intestinal epithelium, regulation the immune system and inflammatory responses, and improving host health [85]. *Faecalibaculum*, also a Gram-positive bacterium of the phylum *Firmicutes*, is one of the most important producers of butyric acid, which has anti-inflammatory properties, maintains bacterial enzyme activity, and protects the digestive system from intestinal pathogens [86]. *Muribaculaceae* is a family of bacteria in the phylum *Mycobacterium*, which produces SCFAs via endogenous (mucinoglycans) and exogenous polysaccharides (dietary fiber) and has a cross-feeding relationship with probiotics such as *Bifidobacterium* and *Lactobacillus* [87]. *Rikenellaceae* is capable of decomposing various organic substances and inhibiting pathogenic microorganisms, which are important for human health [88]. *L. salivarius* NCUH062003 promotes Lactobacillus, Faecalibaculum, Muribaculaceae, Alloprevotella, Alistipes, Dubosiella, Lachnospiraceae, and Rikenellaceae, and other SCFA-producing beneficial bacteria multiply and inhibit the growth of conditionally pathogenic bacteria such as Erysipelothrix, Akkermansia, Bacteroides, ParaBacteroides, Klebsiella, Citrobacter, and Clostridium, thus ameliorating gut microbiota disruptions caused by amoxicillin/metronidazole and *H. pylori*.

Moreover, β-lactams can alter the entire microbiological environment of the gut and the interactions between that microecosystem and the host with great impact when targeted for the therapy of diseases associated with a specific bacterial infection [89]. Glycolysis, pyruvate decarboxylation, tricarboxylic acid cycle, glutamate metabolism, and iron uptake do not return to normal immediately after antibiotic withdrawal [90]. Also, changes in the composition of the gut microbiota as well as metabolic levels are most dramatic on days 6, 11, and 14 of antibiotic use [91]. Similarly, the drug group of this study used amoxicillin belonging to β-lactams and metronidazole belonging to nitroimidazoles for the therapy of HP-infected mice. In the early stages of antibiotic administration, the gut microbiota responds to antibiotic application by the activation of the host stress system, which ‘presumptively’ curtails its total energy metabolism and reduces its substance production to escape the ‘antimicrobial effects’ of the drug application [91]. In this study, continuous therapy with *L. salivarius* NCUH062003 after 14 days of antibiotic administration and 28 days of adjuvant intervention revealed significant recovery in both the biodiversity and abundance of gut microbes in mice.

### 3.7. Effect of Prophylactic Modalities on the Gut Microbiota of HP-Infected Mice

#### 3.7.1. Diversity of Gut Microbiota

**Figure 9:**
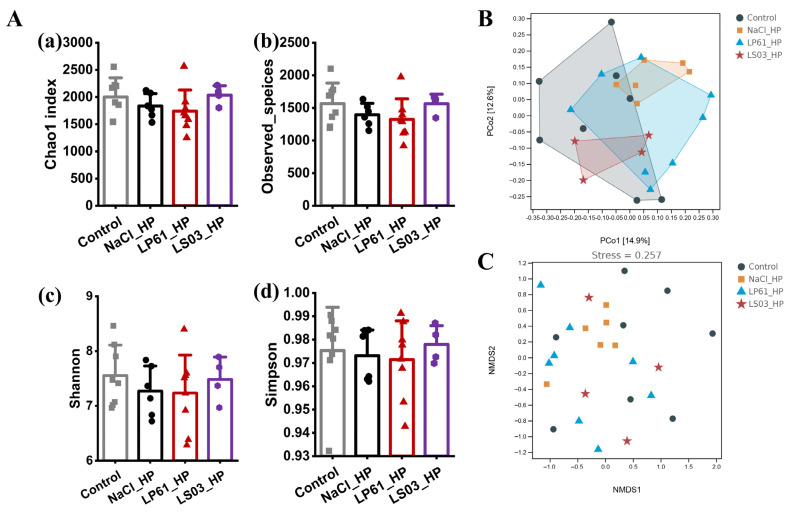
(**A**) Alpha diversity index of the mice gut microbiota samples in each prophylactic group: (**a**) ACE index, (**b**) Chao1 index, (**c**) Shannon index, and (**d**) Simpson index. The beta diversity of gut microbiota of mice in prophylactic groups: (**B**) PCoA plot and (**C**) NMDS plot. Prophylactic groups: control, NaCl_HP, LP61_HP, and LS03_HP groups. A total of 4 groups were formed, with 8 mice in each group.

The effect of *Lactobacillus* in the prophylactic model on the alpha diversity of the HP-infected mice gut microbiota is shown in Figure 9A. The Simpson index of the NaCl_HP group was slightly lower than that of the control group, and the Shannon index, Chao1 index, and observed species index were significantly lower than those of the control group, indicating that a pregavage of 0.9% saline for 4 weeks followed by a gavage of *H. pylori* for 4 weeks in the prophylactic mode had a negative impact on the species diversity and species richness of the mice gut microbiota. However, the Chao1 index, observed species index, Shannon index, and Simpson index in the LS03_HP group were significantly higher than those in the NaCl_HP group (*p* < 0.01) and did not change significantly from those in the control group (*p* > 0.05), whereas those in the LP61 group were lower than those in the NaCl_HP group (*p* < 0.05) and significantly lower than those in the control group (*p* > 0.01). These results indicate that *L. salivarius* NCUH062003 maintained alpha diversity by significantly preventing the species richness and species diversity of the mice gut microbiota affected by *H. pylori*, whereas the control strain, *L. plantarum* LP61, did not prevent the reduction in species richness and species diversity of the gut microbiota of HP-infected mice.

Beta diversity is commonly characterized by means of the downscaling and visualization of microbial data such as PCoA and NMDS based on the Bray–Curtis distance. The effect of *Lactobacillus* or antibiotics in prophylactic mode on the beta diversity and beta diversity distances of the gut microbiota of HP-infected mice are shown in Figure 9B,C and Table S4. PCoA, and the NMDS plots both show that there was a large sample difference distance between the mice gut microbiota in the NaCl_HP and control groups (R = 0.170, *p* = 0.067), and the sample difference distance between the mice gut microbiota in the LS03_HP group and the control group was smaller than that between the NaCl_HP and control group (R = 0.057, *p* = 0.029). The sample difference distance of the gut microbiota of mice between the LP61_HP group and control group was greater than that between the NaCl_HP and control group (R = 0.217, *p* = 0.029), and there was large feature overlap in the sample difference distance matrix confidence intervals between mice in the LS03_HP group and those in the control group, whereas the LS03_HP and LP61_HP groups had the largest beta diversity distance (R = 0.379, *p* = 0.013). The results show that *H. pylori* significantly altered the beta diversity of the gut microbiota of mice and increased the species compositional heterogeneity of the gut microbiota of mice; a 4-week pregavage of mice with *L. salivarius* NCUH062003 prevented the alteration of beta diversity of the gut microbiota by *H. pylori* infection, whereas *L. plantarum* LP61 failed to prevent the alteration of beta diversity. Instead, the pregavage of *L. plantarum* LP61 exacerbated the beta diversity alterations caused by *H. pylori* infection.

#### 3.7.2. Composition of Gut Microbiota

**Figure 10:**
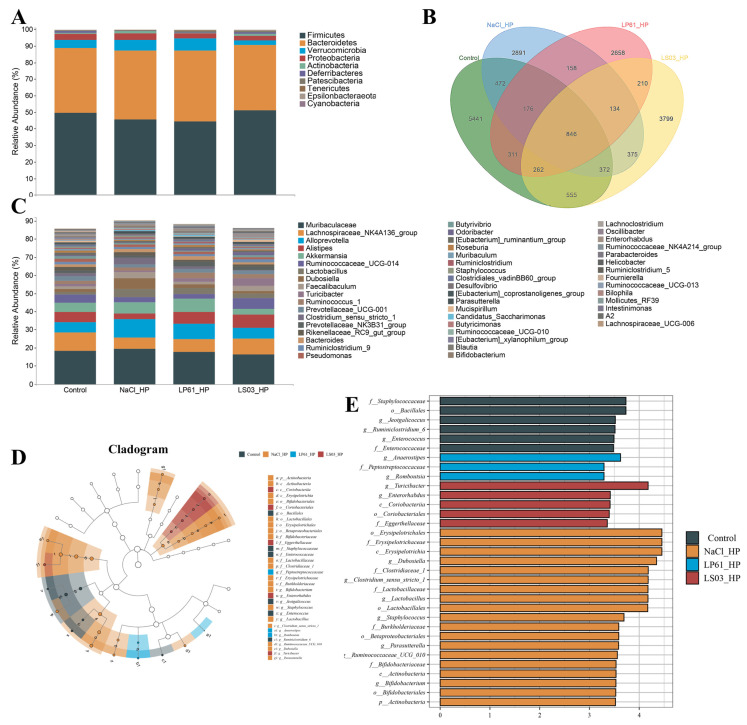
(**A**) The relative abundance of gut microbiota at the phylum level of mice in each prophylactic group. (**B**) Venn diagram of the gut microbiota of mice in each prophylactic group. (**C**) The relative abundance of gut microbiota at the genus level of mice in each prophylactic group. The taxonomic cladogram (**D**) and the histogram (**E**) from LEfSe analysis of the gut microbiota in prophylactic groups. Prophylactic groups: control, NaCl_HP, LP61_HP, and LS03_HP groups. A total of 4 groups were formed, with 8 mice in each group.

As shown in Figure 10B, the number of exclusive ASVs of the gut microbiota in the NaCl_HP group (2891) was much lower than that in the control group (5441). The number of exclusive ASVs of the gut microbiota in the LP61 group (2658) was even lower than that in the NaCl_HP group, whereas the number of exclusive ASVs of the gut microbiota in the LS03 group (3799) was much higher than that in the NaCl_HP group; also, the group that shared the highest number of ASVs with the control group was the LS03_HP group (2035), followed by the NaCl_HP group (1870) and the LP61_HP group (1595). These results indicate that *H. pylori* reduced the number of exclusive species in the mice gut microbiota in the prophylactic model, whereas *L. plantarum* LP61 was unable to alleviate the *H. pylori*-induced reduction in the number of exclusive species in the mice gut microbiota, whereas *L. salivarius* NCUH062003 was able to prevent the decrease in the number of exclusive species in the mice gut microbiota induced by *H. pylori* to a certain extent.

In addition, as shown in Figure 10A and Figure S5, the dominant phyla of the gut microbiota in mice in each prophylactic group were *Bacteroidetes* and *Firmicutes* (relative abundances 39.25–42.81%, 44.29–51.05%). Compared with the control group, the relative abundance of *Firmicutes* was significantly decreased in the NaCl_HP group, while *Bacteroidetes*, *Verrucomicrobia*, and *Proteobacteria* were significantly increased; compared with the NaCl_HP group, in the LP61_HP group, the relative abundance of *Firmicutes* and *Proteobacteria* decreased significantly, while *Bacteroidetes* and *Verrucomicrobia* increased significantly; and compared with the NaCl_HP group, in the LS03_HP group, the relative abundance of *Firmicutes* increased significantly, and *Bacteroidetes*, *Verrucomicrobia*, and *Proteobacteria* decreased significantly. The results show that pretreatment with *L. salivarius* NCUH062003 prevented the decrease in *Firmicutes* and the increase in *Bacteroidetes*, *Proteobacteria*, and *Verrucomicrobia* in the mice gut microbiota caused by *H. pylori* colonization, whereas the control strain *L. plantarum* LP61 did not have a prophylactic effect on component disorders of the gut microbiota.

Furthermore, at the genus level, the relative abundance of the mice gut microbiota in each prophylactic group is shown in Figure 10C and Figure S6. Compared with the control group, in the NaCl_HP group, the relative abundances of *Akkermansia* and *Clostridium* significantly increased, while those of *Lachnospiraceae*, *Alistipes*, *Ruminococcaceae*, *Turicibacter*, *Prevotellaceae*, *Butyrivibrio*, and *Odoribacter* significantly decreased; in the LP61_HP group, the relative abundance of *Akkermansia* did not decrease and was significantly higher than that in the NaCl_HP group; the relative abundances of *Ruminococcaceae* and *Butyrivibrio* did not increase and were not significantly different from that in the NaCl_HP group; in the LS03_HP group, the relative abundances of *Akkermansia* and *Clostridium* were significantly lower than that in the NaCl_HP group and decreased to a level significantly lower than that in the control group; the abundances of *Lachnospiraceae* and *Butyrivibrio* increased significantly but did not recover to the level of control group; the abundance of *Prevotellaceae* increased to a level significantly lower than that of the control group; and the abundances of *Alistipes*, *Ruminococcaceae*, *Turicibacter*, and *Odoribacter* increased significantly and exceeded that of the control group. Even further, as shown in Figure 10D,E, *Jeotgalicoccu*, *Enterococcus*, and *Ruminococcaceae* were significantly enriched in the control group, and *Erysipelothrix* and *Clostridium* were enriched in the NaCl_HP group. *Peptostreptococcus*, Anaerostipes, and Romboutsia were significantly enriched in the LP61_HP group, and *Turicibacter*, enterorhabdus, and *Coriobacteria* were significantly enriched in the LS03_HP group. *Akkermansia*, *Clostridium*, and *Erysipelothrix* can all invade the host independently or in concert with other genera, causing intestinal inflammation and endogenous infections, which can adversely affect the health and even the life of the host [73,75,92]. In contrast, *L. salivarius* NCUH062003 pretreatment intervention prevented, to some extent, the increase in *Akkermansia*, *Clostridium*, and *Erysipelothrix* due to colonization by *H. pylori*, and increased *Lachnospiraceae*, *Alistipes*, *Ruminococcaceae*, *Prevotellaceae*, *Butyrivibrio*, *Odoribacter*, and other producers of short-chain fatty acids (SCFAs) [85], as well as the core gut microbiota such as *Turicibacter* and *Coriobacteria*. *Turicibacter* causes genetic changes that alter bile acid and lipid metabolism in the host [93], and *Coriobacteria* have important functions in the gut for bile salt and steroid conversion and dietary polyphenol activation [94]. In contrast, *L. plantarum* LP61 intervention did not reverse the levels of core intestinal genera, such as *Ruminococcaceae* and *Butyrivibrio*, but increased the abundance of some conditionally pathogenic bacteria, including *Akkermansia* and *Peptostreptococcus*. *Peptostreptococcus* can cause infections in various tissues and organs of the human body and can cause bacterial endocarditis as well as severe traumatic infections [95].

### 3.8. Effects of L. salivarius NCUH062003 on Intestinal Potential Function

**Figure 11:**
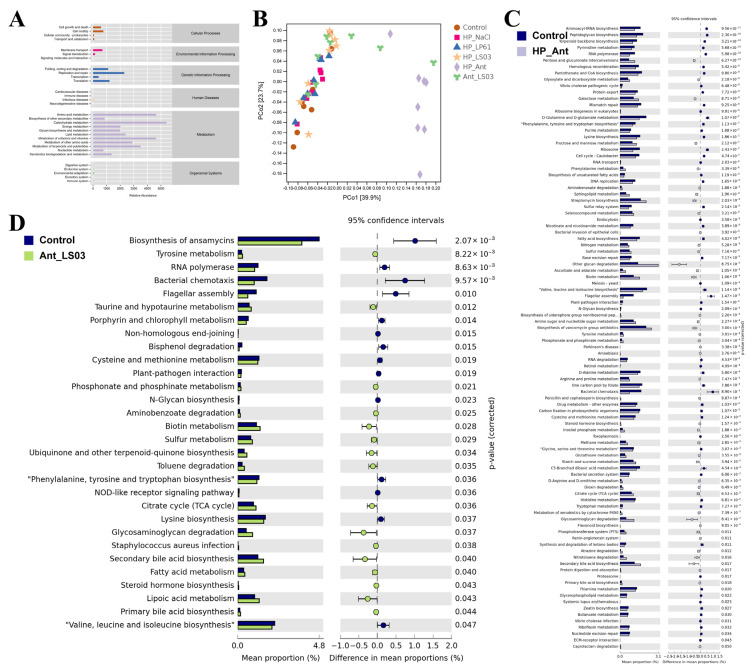
(**A**) Metabolic pathway statistical map for predicted functions of gut microbiota in each therapeutic group. (**B**) PCoA for potential functional units of the gut microbiota of mice in each therapeutic group. (**C**) Significantly different metabolic pathways between the HP_Ant and control groups in the predicted functions of gut microbiota. (**D**) Significantly different metabolic pathways between the Ant_LS03 and control groups in the predicted functions of gut microbiota. Therapeutic groups: control, HP_NaCl, HP_LP61, HP_LS03, HP_Ant, and Ant_LS03 groups. A total of 6 groups were formed, with 8 mice in each group.

As shown in Figure 11B, the functional unit PCoA plot, the results of the predicted functional differences between the groups of samples are similar to those of the gut microbiota. Based on the KEGG metabolic pathway database, the obtained functional units were calculated to derive the abundance values of metabolic pathways, and the results are shown in Figure 11A. The metabolic pathways of the mice gut microbiota in each therapy group fell into six major categories, including cellular processes, environmental information processing, genetic information processing, human diseases, metabolism, and organismal systems. Of these, the metabolic category had the most abundant pathways, including amino acid metabolism, secondary metabolite biosynthesis, carbohydrate metabolism, energy metabolism, polysaccharide biosynthesis and metabolism, lipid metabolism, nucleotide metabolism, and xenobiotic biodegradation and metabolism. To further analyze the effects of *L. salivarius* NCUH062003 and antibiotic therapy on the potential function of the gut microbiota in mice, metabolic pathways that differed significantly between groups were explored.

As shown in Figure 11C, after amoxicillin/metronidazole intervention, there were 98 KOs in the predicted functional metabolic pathways of the gut microbiota in the HP_Ant group versus the control group that were significantly different. Among them, there were amino acid metabolism including alanine, phenylalanine, tryptophan, D-glutamine, and D-glutamate; amino acid biosynthesis including lysine, valine, leucine, isoleucine, phenylalanine, tyrosine, and tryptophan; antibiotic biosynthesis including penicillin, cephalosporin, streptomycin, and vancomycin; and peptidoglycan biosynthesis, fatty acid synthesis, fatty acid metabolism, purine metabolism, and propionic acid metabolism. The results show that antibiotic therapy significantly altered the predicted function of the gut microbiota in HP-infected mice.

As shown in Figure 11D, after 4 weeks of *L. salivarius* NCUH062003-assisted amoxicillin/metronidazole therapy, only 30 KOs of the predicted functional metabolic pathways of the gut microbiota in the Ant_LS03 group versus the control group were significantly different, including the citric acid cycle, biotin metabolism, fatty acid metabolism, tyrosine metabolism, lipoic acid metabolism, and biosynthesis of phenylalanine, tyrosine, and tryptophan. This was significantly fewer than the 98 differential KOs between the HP_Ant group and the control group. The results show that adjuvant therapy with *L. salivarius* NCUH062003 altered the metabolic pathway of the predicted function of the gut microbiota in antibiotic-treated mice and converted it to the normal gut microbiota function in normal mice.

### 3.9. Effects of L. salivarius NCUH062003 on Intestinal SCFAs

Changes in the gut microbiota may lead to changes in gut metabolites [96]. Short-chain fatty acids (SCFAs), as important metabolites of the gut microbiota, are metabolites produced by the fermentation of dietary fiber by beneficial bacteria in the gut microbiome, including acetic, propionic, and butyric acids, which re-uptake unabsorbed carbohydrates and take up water and electrolytes in the colon [97]. SCFAs often protect the gut by disrupting the intestinal osmolality and pH homeostasis, promoting the expression of antimicrobial peptides in the host, affecting the nutrient uptake and energy production of pathogenic bacteria [98], and their working concentrations are well below the threshold of toxicity to host cells [99]. Moreover, research has shown that SCFAs help to promote the recovery of intestinal barrier function in antibiotic-related diarrhea rats [100]. For this study, the levels of SCFAs in the feces of each group are shown in Figure S7.

In the HP_Ant group, the levels of acetic acid, propionic acid, butyric acid, and total SCFAs were significantly lower than those in the control group, suggesting that antibiotic therapy significantly reduced the levels of SCFAs in the gut of HP-infected mice, which may be related to the antibiotic-induced dysbiosis of the gut microbiota of mice in the HP_Ant group. Significant lower species richness and diversity of the gut microbiota and the significant reduction in the potential functions of propionic acid metabolism and butyric acid metabolism contributed to the significant reduction in SCFAs in the HP_Ant group. However, under the intervention of *L. salivarius* NCUH062003, the decreasing trend of SCFAs was reversed. In the Ant_LS03 group, the contents of acetic acid and total SCFAs were significantly higher than those in the HP_Ant group, and even the contents of acetic, propionic, and butyric acids were significantly higher than those in the control group, which may be related to the restoration of the gut microbiota diversity in antibiotic-containing mice by NCUH062003, as well as a significant increase in the abundance of SCFAs-producing bacteria, mainly *Lactobacillus*. However, ingestion of *L. gasseri* LG21, the commercial anti-*H. pylori* strain, only resulted in an increase in isobutyric acid in the gut [101]. SCFAs regulate the function of natural immune cells involved in the immune system, e.g., macrophages, neutrophils, and dendritic cells, as well as the differentiation of T and B cells and antigen-specific adaptive immunity [85]. SCFAs as metabolites of the intestinal microbiota resulted in trends similar to those of the intestinal microbiota predicting functional metabolic pathways. SCFA metabolic pathways and their immunological role contribute to the restoration of gut microbiota diversity.

## 4. Conclusions

In this study, models of probiotic prophylaxis and therapy of *H. pylori* infection in C57BL/6 mice were established. We found that *L. salivarius* NCUH062003 could attenuate the damage caused by amoxicillin/metronidazole to the liver of mice and reduce the colonization of *H. pylori* in the gastric mucosa of mice by inhibiting *H. pylori* growth and suppressing the urease activity. Importantly, NCUH062003 was able to reduce the expression of inflammatory factors and myeloperoxidase (MPO), attenuate *H. pylori*-mediated neutrophil chemotaxis, and alleviate the hemorrhagic damage of the gastric mucosa muscularis propria. Meanwhile, NCUH062003 had an anti-oxidative stress effect, resisting the apoptosis of gastric epithelial cells, increasing the level of superoxide dismutase (SOD), and promoting the expression of the tight junction protein ZO1 and the membrane integrative protein Occludin, which can restore the damaged gastric mucosa. In addition, in the therapy mode, NCUH062003 restored alpha diversity, mitigated beta diversity alterations, and decreased the number of exclusive species of the mice gut microbiota, significantly alleviating the negative effects caused by *H. pylori* colonization and amoxicillin/metronidazole treatment. Also, in the prophylactic mode, the 4-week pretreatment intervention of NCUH062003 somewhat prevented the increase in *Clostridium*, *Akkermansia*, and *Erysipelothrix* and increased the levels of SCFA producers including *Ruminococcaceae*, *Prevotellaceae*, *Butyrivibrio*, and *Odoribacter* and the gut core microbiota including *Turicibacter* and *Coriobacteria*. Furthermore, NCUH062003 adjuvant amoxicillin/metronidazole therapy altered the metabolic pathways of the predicted function of the gut microbiota in antibiotic therapy mice, converged to normal mice, and restored the levels of SCFAs to varying degrees. In conclusion, compared with antibiotic therapy and existing commercial probiotics such as *L. gasseri* LG21 and *L. johnsonii* No. 1088, etc., NCUH062003 as both a preventive and therapeutic agent efficiently inhibited *H. pylori*, attenuated the inflammatory response, promoted mucosal repair, and alleviated gut microbiota disorders, which could help to both prevent *H. pylori* infection and develop antibiotic-probiotic combination therapy. In the future, we expect to conduct other mouse lines and human trials, analyzing the improvement effect of NCUH062003 on patients with *H. pylori*. Meanwhile, we hope to expand on potential clinical applications, e.g., preparing NCUH062003 bacteriological agents and addressing scalability for human trials and long-term effects.

## Figures and Tables

**Table 1:** Body weight (g) and organ coefficient (mg/g).

Groups	Body Weight (g)	Organ Coefficient (mg/g)				
	Day 70	Heart	Liver	Spleen	Lung	Kidney
Control	30.77 ± 1.67 ^a^	4.44 ± 0.60 ^a^	37.71 ± 3.95 ^a^	2.60 ± 0.70 ^a^	4.68 ± 0.56 ^a^	10.68 ± 0.68 ^a^
HP_NaCl	29.34 ± 1.45 ^a^	5.19 ± 1.51 ^ab^	33.64 ± 2.98 ^b^	2.54 ± 0.29 ^a^	4.87 ± 0.70 ^a^	9.92 ± 0.57 ^ab^
HP_LP61	29.91 ± 1.41 ^a^	5.70 ± 1.05 ^ab^	34.27 ± 3.34 ^ab^	2.47 ± 0.21 ^a^	5.19 ± 0.77 ^a^	10.68 ± 1.11 ^a^
HP_LS03	29.61 ± 2.41 ^a^	5.61 ± 1.14 ^ab^	35.97 ± 2.90 ^ab^	2.47 ± 0.51 ^a^	5.21 ± 1.08 ^a^	9.89 ± 0.48 ^ab^
HP_Ant	29.86 ± 1.30 ^a^	5.28 ± 0.91 ^ab^	33.32 ± 2.67 ^b^	2.41 ± 0.34 ^a^	5.03 ± 0.43 ^a^	10.51 ± 0.75 ^a^
Ant_LS03	28.48 ± 1.53 ^ab^	5.92 ± 1.00 ^b^	35.85 ± 3.35 ^ab^	2.67 ± 0.60 ^a^	5.47 ± 0.71 ^ab^	10.85 ± 1.36 ^a^
NaCl_HP	29.77 ± 1.34 ^a^	6.06 ± 1.99 ^b^	32.09 ± 2.20 ^b^	2.05 ± 0.71 ^ab^	5.21 ± 1.17 ^a^	10.23 ± 1.66 ^a^
LP61_HP	30.36 ± 2.53 ^a^	5.18 ± 1.28 ^ab^	32.64 ± 2.41 ^b^	2.24 ± 0.28 ^ab^	5.32 ± 0.75 ^a^	10.80 ± 1.15 ^a^
LS03_HP	30.08 ± 1.15 ^a^	5.07 ± 0.60 ^ab^	34.48 ± 2.93 ^ab^	2.30 ± 0.29 ^ab^	4.75 ± 0.80 ^a^	9.81 ± 0.67 ^ab^

Different lowercase letters in a column indicate significant differences (*p* < 0.05).

## Data Availability

The original contributions presented in this study are included in the article/Supplementary Material. Further inquiries can be directed to the corresponding author.

## Supplementary Materials

The following supporting information can be downloaded at: https://www.mdpi.com/article/10.3390/microorganisms12122521/s1, Figure S1: (A) Histopathology of heart, liver, spleen, lung, and kidney tissue of mice from different groups by hematoxylin and eosin staining (200×). (B) Changes of body weight in each mice group; Figure S2: Evaluation of *H. pylori* infection in mice by urease assay (a), H&E staining (b), and plate coating (c) of gastric tissues; Figure S3: The relative abundance of *Bacteroidetes* (A), *Firmicutes* (B), *Verrucomicrobia* (C), and *Proteobacteria* (D) of gut microbiota in mice of each therapeutic mouse group; Figure S4: The relative abundance of *Akkermansia* (A), *Bacteroides* and *Parabacteroides* (B), *Klebsiella* (C), *Lachnospiraceae* (D), *Alloprevotella* (E), *Alistipes* (F), *Dubosiella* (G), *Lactobacillus* (H), and *Faecalibaculum* (I) of gut microbiota in mice of each therapeutic group; Figure S5: The relative abundance of *Bacteroidetes* (A), *Firmicutes* (B), *Verrucomicrobia* (C), and *Proteobacteria* (D) of gut microbiota in mice of each prophylactic mice group; Figure S6: The relative abundance of *Akkermansia* (A), *Clostridium* (B), *Lachnospiraceae* (C), *Alistipes* (D), *Ruminococcaceae* (E), *Turicibacter* (F), *Prevotellaceae* (G), *Butyrivibrio* (H), and *Odoribacter* (I) of gut microbiota in mice of each prophylactic group; Figure S7: The levels of acetate (A), propionate (B), butyrate (C), and total SFCAs (D) in the feces of *H. pylori* mice in each group; Table S1: Determination of the urease activity of the mouse gastric mucosa in therapeutic and prophylactic groups; Table S2: Histopathological outcomes of gastric antrum tissue in the pyloric part of mice from different groups; Table S3: Intergroup difference analysis of intestinal flora of mice in *H. pylori* therapeutic groups; Table S4: Intergroup difference analysis of intestinal flora of mice in *H. pylori* prophylactic groups.
