# Supplementary material for: Helicobacter pylori Inhibition, Gastritis Attenuation, and Gut Microbiota Protection in C57BL/6 Mice by Ligilactobacillus salivarius NCUH062003
Source: Microorganisms. 2024 Dec 7;12(12):2521. doi: 10.3390/microorganisms12122521 (PMC11678540; doi:10.3390/microorganisms12122521)
Supplement: Supplementary file 1 [file microorganisms-12-02521-s001.zip › microorganisms-3315715-supplementary.pdf]

Figures

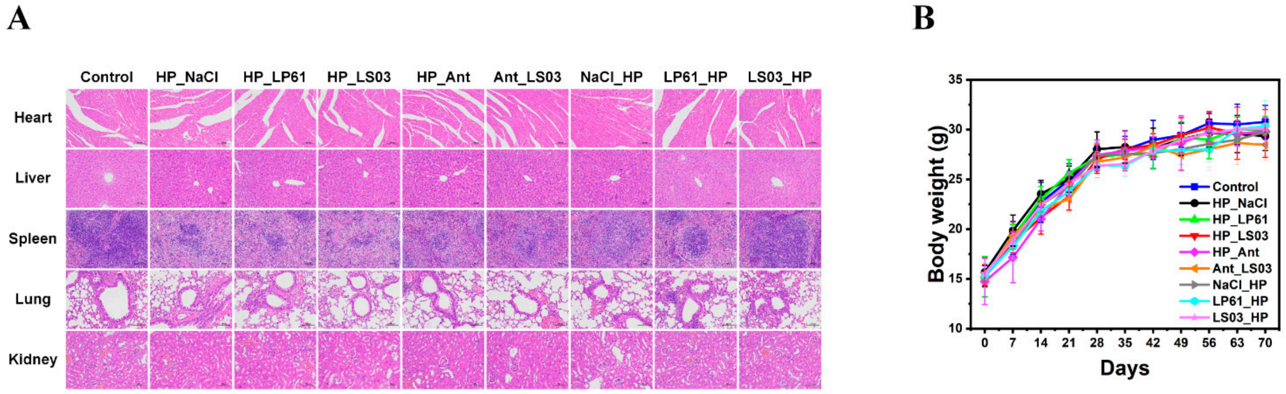

Figure S1. (A) Histopathology of heart, liver, spleen, lung, and kidney tissue of mice from different groups by hematoxylin and eosin staining (200 ×). (B) Changes of body weight in each mice group. Mice experiments lasted for 70 days.

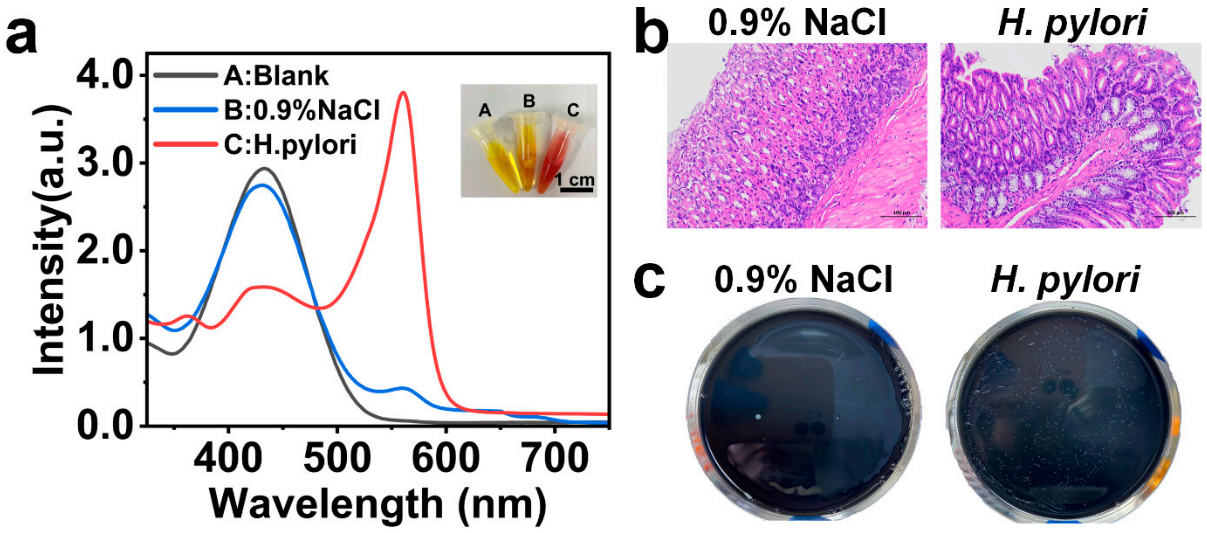

Figure S2. Evaluation of *H. pylori* infection in mice by urease assay (a), H&E staining (b), and plate coating (c) of gastric tissues.

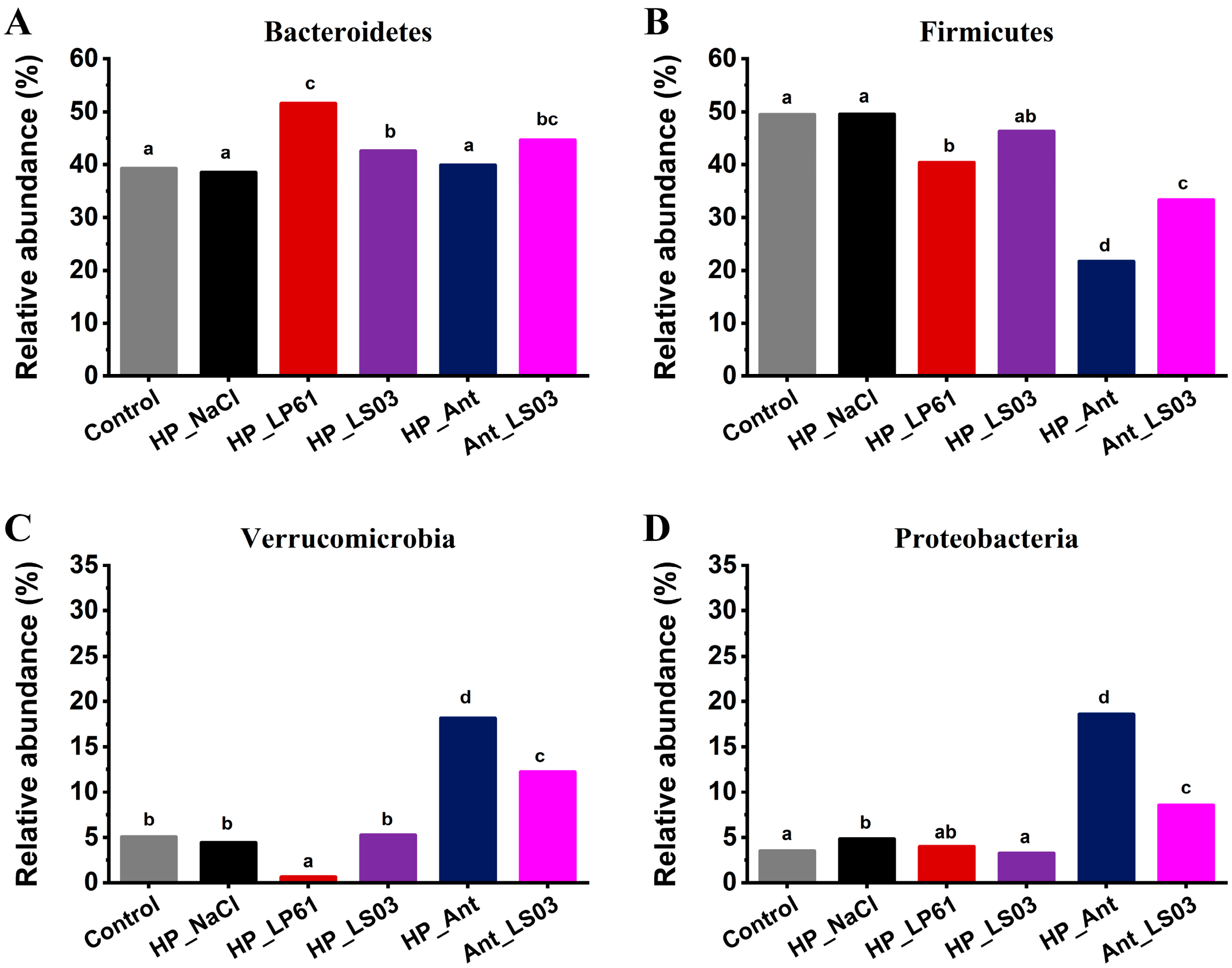

Figure S3. The relative abundance of *Bacteroidetes* (A), *Firmicutes* (B), *Verrucomicrobia* (C), and *Proteobacteria* (D) of gut microbiota in mice of each therapeutic mouse group.

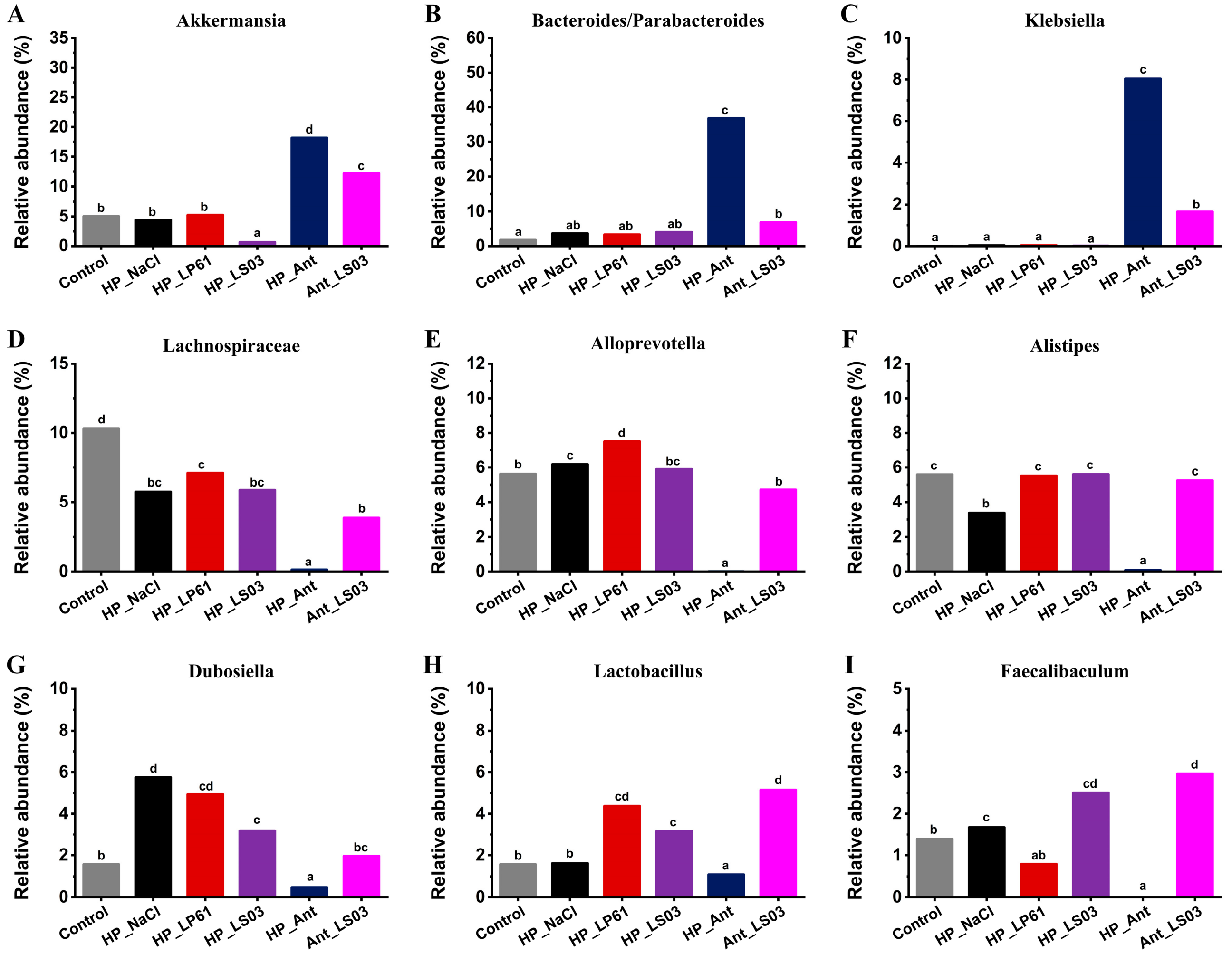

Figure S4. The relative abundance of *Akkermansia* (A), *Bacteroides* and *Parabacteroides* (B), *Klebsiella* (C), *Lachnospiraceae* (D), *Alloprevotella* (E), *Alistipes* (F), *Dubosiella* (G), *Lactobacillus* (H), and *Faecalibaculum* (I) of gut microbiota in mice of each therapeutic group

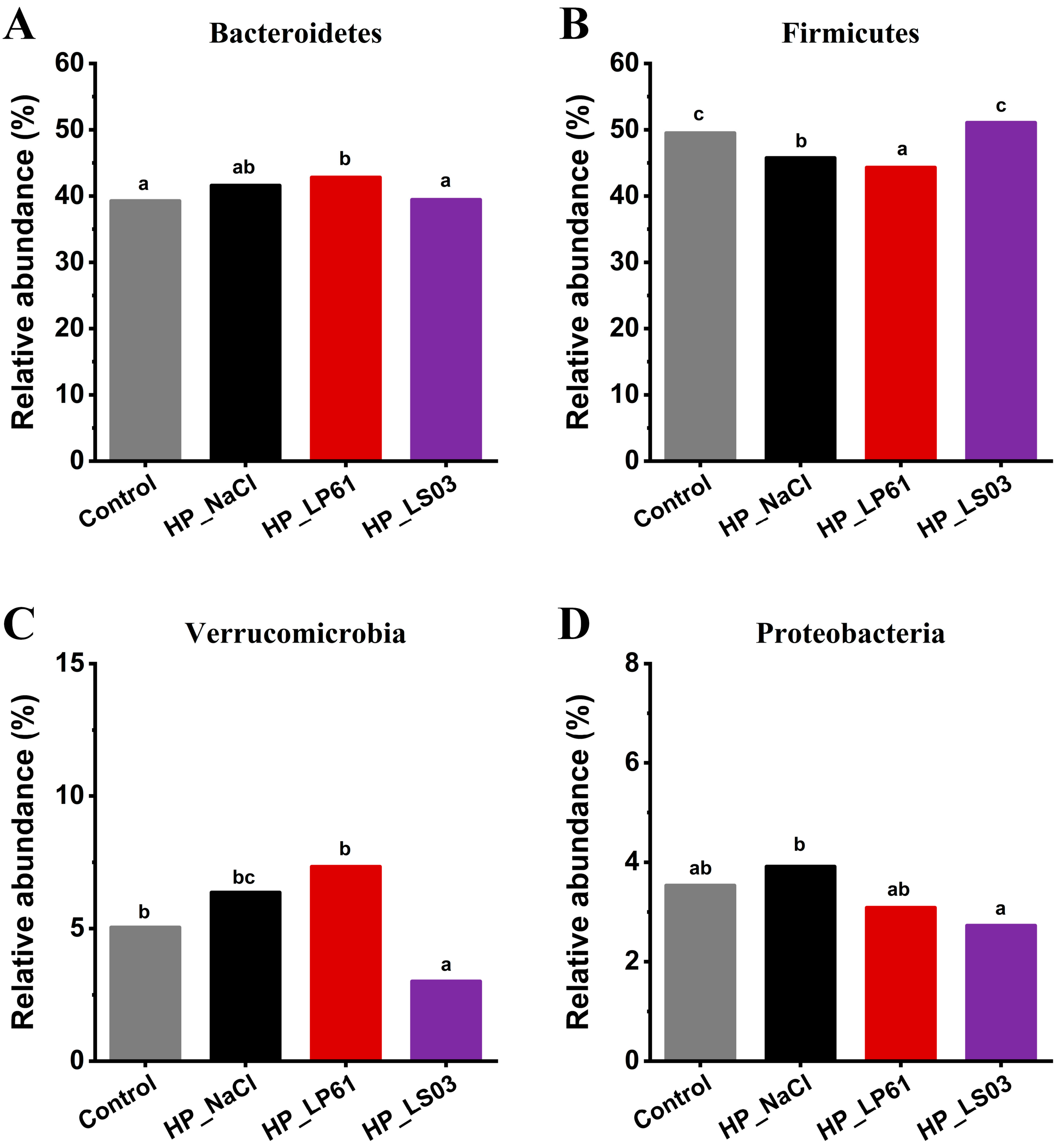

Figure S5. The relative abundance of *Bacteroidetes* (A), *Firmicutes* (B), *Verrucomicrobia* (C), and *Proteobacteria* (D) of gut microbiota in mice of each prophylactic mice group.

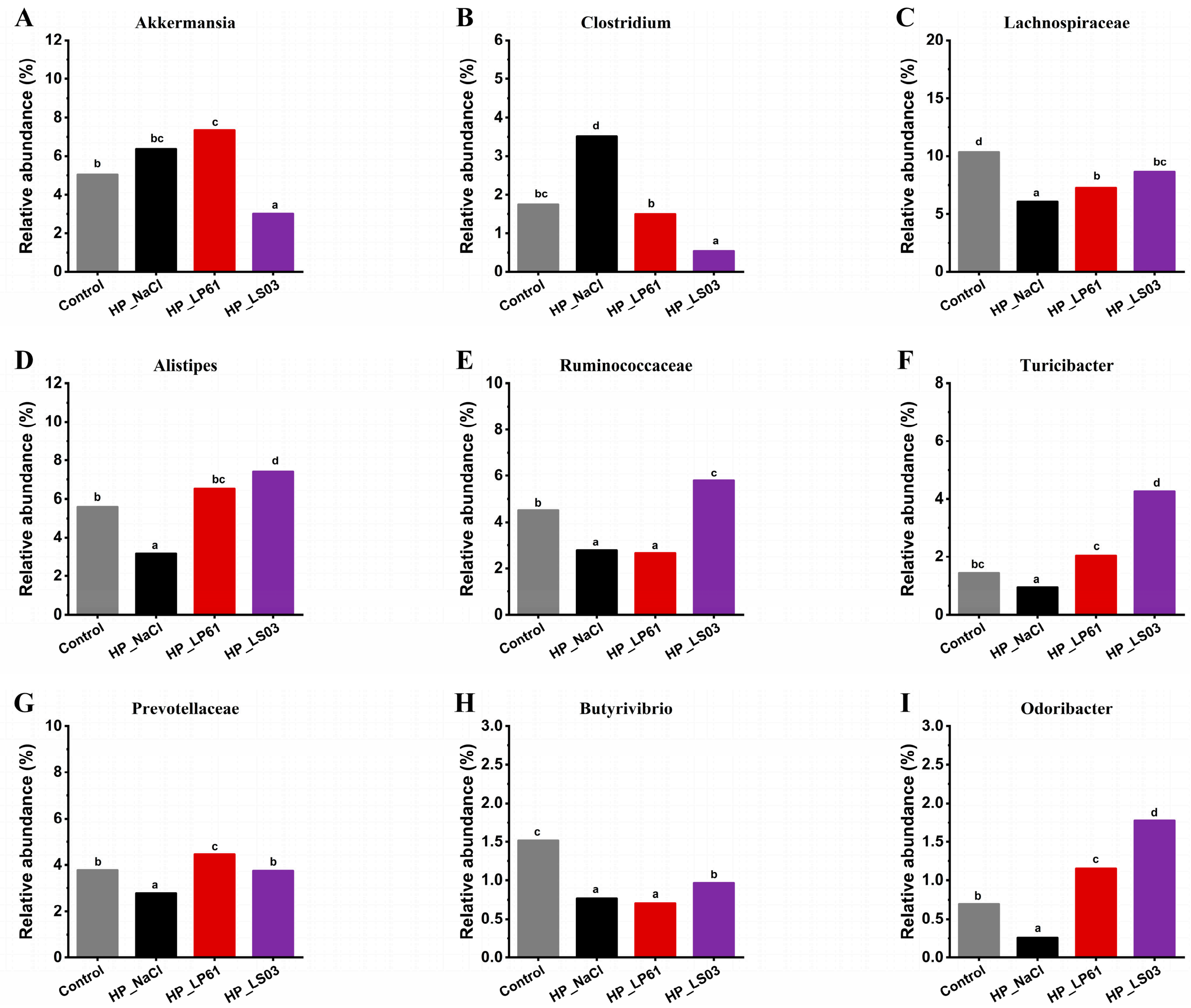

Figure S6. The relative abundance of *Akkermansia* (A), *Clostridium* (B), *Lachnospiraceae* (C), *Alistipes* (D), *Ruminococcaceae* (E), *Turicibacter* (F), *Prevotellaceae* (G), *Butyrivibrio* (H), and *Odoribacter* (I) of gut microbiota in mice of each prophylactic group.

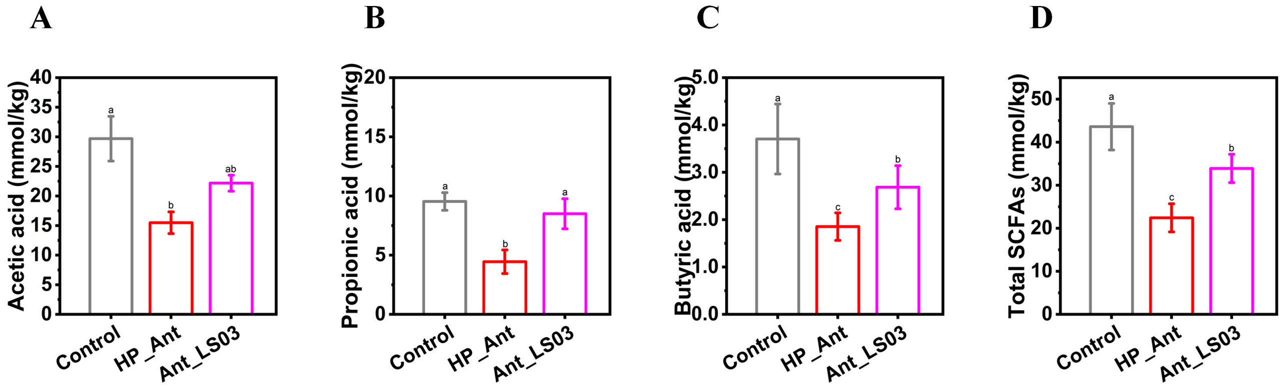

Figure S7. The levels of acetate (A), propionate (B), butyrate (C), and total SFCAs(D) in the feces of *H. pylori* mice in each group.

Tables

Table S1. Determination of the urease activity of the mouse gastric mucosa in therapeutic and prophylactic groups.

Group	Control	HP_NaCI	HP_LP61	HP_LS03	HP_Ant	Ant_LSO3	NaCl_HP	LP61_HP	LSO3_HP
Urease test solution (OD)	0.27 ± 0.11	3.91 ± 0.97	3.75 ± 0.86	1.78 ± 0.79	0.66 ± 0.42	0.39 ± 0.18	4.59 ± 0.66	2.84 ± 0.35	1.44 ± 0.26

Part of the gastric antrum tissue was homogenized to detect urease content by a modified phenol red method. In brief, the gastric tissue homogenates were incubated with the urea-phenol red solution (20% urea and 0.012% phenol, pH 6.5) for 1 h, and the OD values were measured at 561 nm by a microplate reader. Data are mean ± standard deviation (n = 8).

.

Table S2. Histopathological outcomes of gastric antrum tissue in the pyloric part of mice from different groups.

Category	Histopathological diagnosis	n (%)[a]								
		Control	HP_NaCI	HP_LP61	HP_LS03	HP_Ant	Ant_LSO3	NaCl_HP	LP61_HP	LSO3_HP
Inflammation lesions	fewer inflammatory cells, confined to the superficial mucosal layer	5(62.5)	0(0)	0(0)	1(12.5)	3(37.5)	3(37.5)	0(0)	0(0)	1(12.5)
	denser inflammatory cells, more than 1/3 of the mucosal layer	3(37.5)	0(0)	0(0)	5(62.5)	3(37.5)	4(50.0)	0(0)	1(12.5)	3(37.5)
	dense inflammatory cells, occupying the whole mucosal layer	0(0)	1(12.5)	3(37.5)	2(25.0)	1(12.5)	1(12.5)	2(25.0)	3(37.5)	4(50.0)
	few neutrophils infiltrated in the lamina propria	0(0)	3(37.5)	3(37.5)	0(0)	1(12.5)	0(0)	3(37.5)	4(50.0)	0(0)
	more neutrophils infiltrated in the lamina propria	0(0)	3(37.5)	2(25.0)	0(0)	0(0)	0(0)	2(25.0)	0(0)	0(0)
	denser neutrophils in the lamina propria and visible small concave abscesses	0(0)	1(12.5)	0(0)	0(0)	0(0)	0(0)	1(12.5)	0(0)	0(0)
Mucosal damage lesions	normal mucosa	7(87.5)	0(0)	0(0)	4(50.0)	0(0)	4(50.0)	0(0)	0(0)	5(62.5)
	damaged epithelial cells	1(12.5)	2(25.0)	5(62.5)	3(37.5)	4(50.0)	4(50.0)	2(25.0)	6(75.0)	3(37.5)
	damaged glandular cells	0(0)	6(75.0)	3(37.5)	1(12.5)	2(25.0)	0(0)	6(75.0)	2(25.0)	0(0)
	vesicles, hemorrhages and ulcers in the mucosal layer	0(0)	0(0)	0(0)	0(0)	2(25.0)	0(0)	0(0)	0(0)	0(0)

[a] n means the number of mice in each group presenting the corresponding histopathological diagnosis status; (%) means the percentage of that number. There were 9 groups, and the number of mice in each group was 8.

Table S3. Intergroup difference analysis of intestinal flora of mice in *H. pylori* therapeutic groups.

Group1	Group2	R	p-value	q-value
all	-	0.553125	0.001	-
Control	HP_NaCl	0.389788	0.001	0.00125
Control	HP_LP61	0.272321	0.008	0.008
Control	HP_LS03	0.352121	0.001	0.00125
Control	HP_Ant	0.97154	0.001	0.00125
Control	Ant_LS03	0.435826	0.001	0.00125
HP_NaCl	HP_LP61	0.273158	0.001	0.00125
HP_NaCl	HP_LS03	0.44615	0.001	0.00125
HP_NaCl	HP_Ant	0.979911	0.001	0.00125
HP_NaCl	Ant_LS03	0.506138	0.001	0.00125
HP_LP61	HP_LS03	0.371652	0.001	0.00125
HP_LP61	HP_Ant	0.991629	0.002	0.002143
HP_LP61	Ant_LS03	0.380022	0.001	0.00125
HP_LS03	HP_Ant	0.985491	0.001	0.00125
HP_LS03	Ant_LS03	0.447545	0.001	0.00125
HP_Ant	Ant_LS03	0.703125	0.002	0.002143

Analysis of similarities (Anosim) non-parametric test was used to discriminate between-group variation and to calculate the test statistic's R-value. The test statistic R takes values between -1 and 1. Less than 0 indicates that the within-group difference is greater than the between-group difference and greater than 0 indicates that the between-group difference is greater than the within-group difference.

Table S4. Intergroup difference analysis of intestinal flora of mice in *H. pylori* prophylactic groups.

Group1	Group2	R	p#value	q#value
all	-	0.253613	0.001	-
Control	NaCl_HP	0.170543	0.067	0.0804
Control	LP61_HP	0.216797	0.029	0.0435
Control	LS03_HP	0.056985	0.029	0.029
NaCl_HP	LP61_HP	0.299903	0.012	0.026
NaCl_HP	LS03_HP	0.75	0.005	0.026
LP61_HP	LS03_HP	0.378676	0.013	0.026

Analysis of similarities (Anosim) non-parametric test was used to discriminate between-group variation and to calculate the test statistic's R-value. The test statistic R takes values between -1 and 1. Less than 0 indicates that the within-group difference is greater than the between-group difference and greater than 0 indicates that the between-group difference is greater than the within-group difference.
